# Neuronal and astrocytic sodium-calcium exchanger differentially regulates calcium and sodium overload during ischemic stroke

**DOI:** 10.1172/jci.insight.200411

**Published:** 2026-06-02

**Authors:** Somayyeh Hamzei Taj, Pawan Kumar Thapaliya, Cordula Rakers, Niklas J. Gerkau, Christine R. Rose, Ghanim Ullah, Gabor C. Petzold

**Affiliations:** 1 Vascular Neurology Research Group, German Center for Neurodegenerative Diseases (DZNE), Bonn, Germany.; 2 Department of Physics, University of South Florida, Tampa, Florida, USA.; 3 Institute of Neurobiology, Heinrich Heine University Düsseldorf, Düsseldorf, Germany.; 4 Department of Vascular Neurology, University Hospital Bonn, Bonn, Germany.

**Keywords:** Neuroscience, Vascular biology, Stroke

## Abstract

Spreading depolarizations (SDs) are propagating waves of near-complete breakdown of transmembrane ion gradients that occur during acute ischemic stroke and worsen outcome by driving calcium overload and glutamate release in neurons and astrocytes. The plasmalemmal sodium-calcium exchanger (NCX) plays a key role in such changes, in that the complex ionic disequilibrium during ischemia induces reverse-mode activity of NCX, leading to cellular calcium overload in exchange for sodium. However, the cell-type-specific roles of NCX in neurons and astrocytes during SDs remain unclear. Here, we used ion and glutamate reporters in an in vivo stroke model in mice carrying inducible, cell-specific deletions of NCX isoform 1. Neuronal NCX1 deletion reduced neuronal and astrocytic calcium transients, increased neuronal sodium transients, decreased extracellular glutamate levels, and raised SD initiation threshold. In contrast, astrocytic NCX1 deletion increased sodium transients in both neurons and astrocytes, and increased neuronal calcium as well as extracellular glutamate levels. A computational model of ischemia confirmed that these effects are consistent with reverse-mode NCX1 activity. Together, these findings indicate opposing roles of reverse-mode NCX1 during ischemia. Neuronal NCX1 promotes SD susceptibility, calcium overload, and glutamate release, whereas astrocytic NCX1 exerts protective effects by attenuating glutamate elevation and neuronal calcium accumulation.

## Abstract 2

During experimental stroke, a calcium-transport protein has opposite effects in brain cells: harmful in neurons but protective in astrocytes, influencing damaging brain activity and recovery.

## Introduction

Stroke is a common and severe neurological disorder caused by the acute cessation of local blood flow in the central nervous system. The infarct core is surrounded by a dynamic peri-infarct tissue zone, called the penumbra, in which cerebral blood flow (CBF) is substantially reduced (1). The current concept of the development and growth of ischemic lesions in the gray matter is largely based on spreading depolarizations (2), which are (initially reversible) waves of near-complete breakdown of transmembrane ion gradients that are associated with cytotoxic edema and precede neuronal damage in the ischemic core and penumbra by more than 10 minutes (3). Further spreading depolarizations usually occur in the following hours to days due to energy supply-demand mismatch in the ischemic penumbra. Potential contributing factors in this context include, for example, the excessive release of depolarizing agents such as extracellular potassium and glutamate (3). Because tissue repolarization after spreading depolarizations is energy consuming, they accelerate neuronal death and injury growth as shown in preclinical models as well as in clinical studies (4–7). Hence, a better understanding of the mechanisms underlying spreading depolarizations in injured but potentially salvageable brain tissue may hold great promise to mitigate secondary neurodegeneration and improve outcome in the wake of ischemia. One such detrimental mechanism is an acute overload of neurons and astrocytes with calcium and sodium during spreading depolarizations. We have previously shown that a large fraction of astroglial calcium elevations during spreading depolarization waves after experimental stroke are mediated by inositol triphosphate receptor type 2–dependent (IP_3_R2-dependent) release from internal calcium stores (8). Moreover, these calcium changes in astrocytes also contributed to a secondary detrimental calcium overload in neurons, likely by calcium-dependent release of glutamate from astrocytes (8).

In addition to the highly relevant involvement of intracellular stores in calcium elevations, a substantial part of spreading depolarization-related calcium signals appears to be gated through other pathways, such as membrane channels or transporters. We previously found that the plasmalemmal sodium-calcium exchanger (NCX) plays a key role in calcium and sodium elevations after stroke. Under physiological conditions, NCX contributes to calcium homeostasis by shuttling out 1 calcium ion in exchange for 3 Na^+^ ions (9). However, NCX can reverse its action when intracellular sodium levels rise, so that reverse-mode NCX may contribute to a detrimental calcium increase under these conditions (10, 11). This is the case in cerebral ischemia, where dysfunction of the Na^+^/K^+^-ATPase induced by energy deprivation leads to sodium elevations in the penumbral tissue (12, 13). Consequently, deletion or inhibition of NCX leads to lower calcium influx into neurons in experimental ischemia (14, 15). Moreover, we have shown that inhibition of NCX indeed amplifies sodium transients and reduces calcium transients during spreading depolarizations in models of chemical hypoxia or experimental ischemia, in line with a reverse-mode action of NCX during spreading depolarizations (16).

However, several open questions remain. First, NCX is widely expressed by excitatory neurons as well as astrocytes in cerebral cortex (17), but the individual contributions of NCX in each cell type to calcium and sodium changes during spreading depolarizations are unclear. This is important, as ion handling profoundly differs between neurons and glia, and hence it has remained unknown whether NCX operates in the reverse mode in both cell types and what the individual consequences are for intracellular ion changes. Secondly, sodium and calcium changes both shape and execute the astroglial and neuronal release and re-uptake of glutamate (18), but the role of NCX in astrocytes and neurons for glutamate homeostasis during spreading depolarizations has remained elusive.

Therefore, we here examined the cell-specific roles of NCX in calcium, sodium, and glutamate changes during cortical spreading depolarizations, using in vivo models of stroke as well as computational models of cerebral hypoxia.

## Results

### Efficient and selective deletion of NCX1 in neurons or astrocytes.

**Figure 1:**
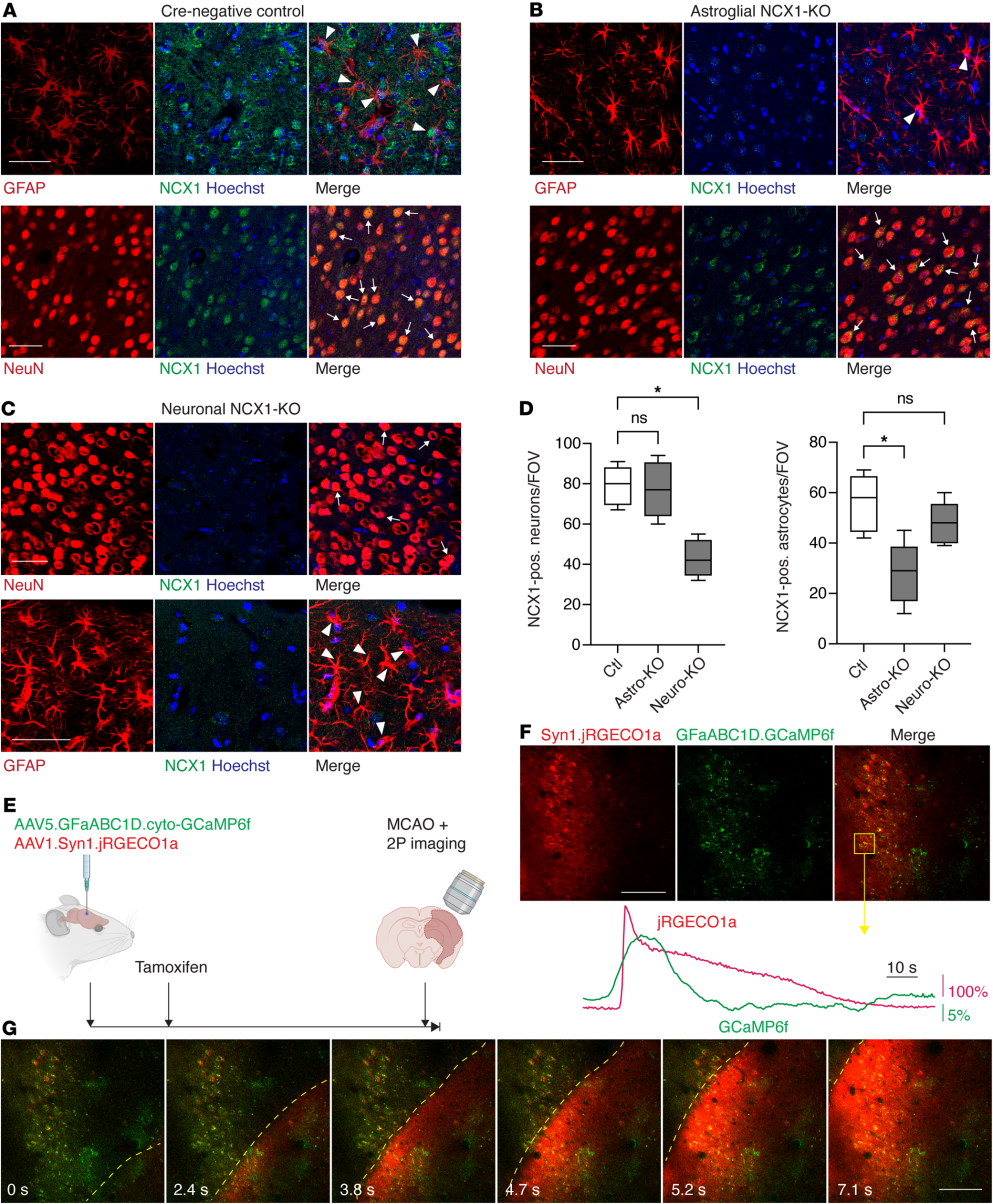
Cell-specific NCX1 deletion and experimental paradigm. (**A**) Immunohistochemistry using antibodies against NCX1, astrocytes (glial fibrillary acidic protein [GFAP]), and neurons (NeuN) showed that NCX1 is expressed by astrocytes (arrowheads) and neurons (arrows) in the cortex of Cre-negative control mice. (**B**) In astroglial NCX1-KO mice, most astrocytes appeared NCX1-negative, although punctuate signals remained in few astrocytes (arrowheads); neuronal NCX1 expression remained unchanged (arrows). (**C**) In neuronal NCX1-KO mice, neuronal NCX1 coverage was attenuated, although few neurons remained NCX1-positive (arrows); astroglial NCX1 coverage appeared unchanged (arrowheads). (**D**) Quantitative analysis of Cre-negative control mice, astroglial NCX1-KO mice, and neuronal NCX1-KO mice. NCX1-positive neurons or astrocytes, respectively, per field of view (FOV) were compared in the 3 lines. Astroglial coverage was significantly attenuated in astroglial NCX1-KO mice, and neuronal coverage was significantly attenuated in neuronal NCX1-KO mice (5 FOVs were averaged per animal; *n* = 5 animals per group; *P < 0.05, Kruskal-Wallis test followed by Dunn’s multiple-comparison test for all comparisons). (**E**) AAV5.GfaABC1D.cyto-GCaMP6f, and AAV1.syn1.jRGECO1a were co-injected into cortex to label astrocytes and neurons, respectively, with calcium indicators. One week later, tamoxifen was administered for Cre activation, followed by middle cerebral artery occlusion (MCAO) and 2-photon microscopy 4 weeks later. (**F** and **G**) During MCAO, ischemia-induced spreading depolarizations occurred, which were characterized by large, slowly progressing, transient calcium elevation in cortical neurons and astrocytes. Scale bars: 50 μm (**A**–**C**) and 100 μm (**F** and **G**).

NCX isoform 1 (NCX1) is widely expressed in the brain, including in pyramidal neurons and astrocytes in the cortex (17). To achieve NCX1 deletion selective for these cell types, we crossed *Camk2a*-CreERT2 mice (19) or *Glast*-CreERT mice (20), respectively, with NCX1-loxP mice (21). Conditional NCX1 deletion was induced by tamoxifen injection 4 weeks prior to experiments (Cre-negative NCX1-loxP mice injected with tamoxifen were used as controls). By immunohistochemistry, we confirmed that NCX1 expression was present in cortical neurons as well as in the neuropil in Cre-negative control mice (Figure 1A). This neuropil signal likely contains neuronal dendrites and glial cells, but using an astrocyte-specific antibody, we confirmed that astrocytes also express NCX1 (Figure 1A), as previously reported (17).

In *Glast*-CreERT:NCX1-loxP (i.e., astroglial NCX1-KO) mice, the NCX1 signal was diminished in astrocytes, but still intact in neurons (Figure 1B). In turn, *Camk2a*-CreERT2:NCX1-loxP (i.e., neuronal NCX1-KO) mice showed an attenuated NCX1 signal in neurons, whereas levels in astrocytes remained unaffected (Figure 1C). A quantification of immunohistochemical signals in both knockout (KO) lines compared with control mice confirmed that neuronal NCX1-KO mice showed a significant (~50%) reduction in neuronal coverage (but unchanged astroglial coverage), and vice versa, astroglial NCX1-KO mice displayed a significant (~45%) reduction in astroglial coverage (but unchanged neuronal coverage), confirming the efficacy and selectivity of our NCX1 deletion strategy (Figure 1D).

### Cell-specific NCX1 deletion differentially modulates spreading depolarization–associated calcium changes in neurons and astrocytes during ischemia.

One week prior to tamoxifen-induced Cre activation, we injected AAV5.GfaABC1D.cyto-GCaMP6f and AAV1.syn1.jRGECO1a together into the cortex to simultaneously record calcium activity from astrocytes and neurons, followed by chronic cranial window implantation (Figure 1, E and F).

We induced focal ischemic stroke by permanent middle cerebral artery occlusion (MCAO) 4 weeks after Cre-mediated NCX1 deletion, and recorded astroglial and neuronal calcium activity immediately after ischemia induction using multiphoton microscopy (Figure 1, E and F). Both indicators reliably reported calcium changes during stroke-induced spreading depolarizations, which were characterized by a large, slowly progressing, transient calcium elevation in cortical neurons and astrocytes (Figure 1, F and G), as previously reported (8, 22).

**Figure 2:**
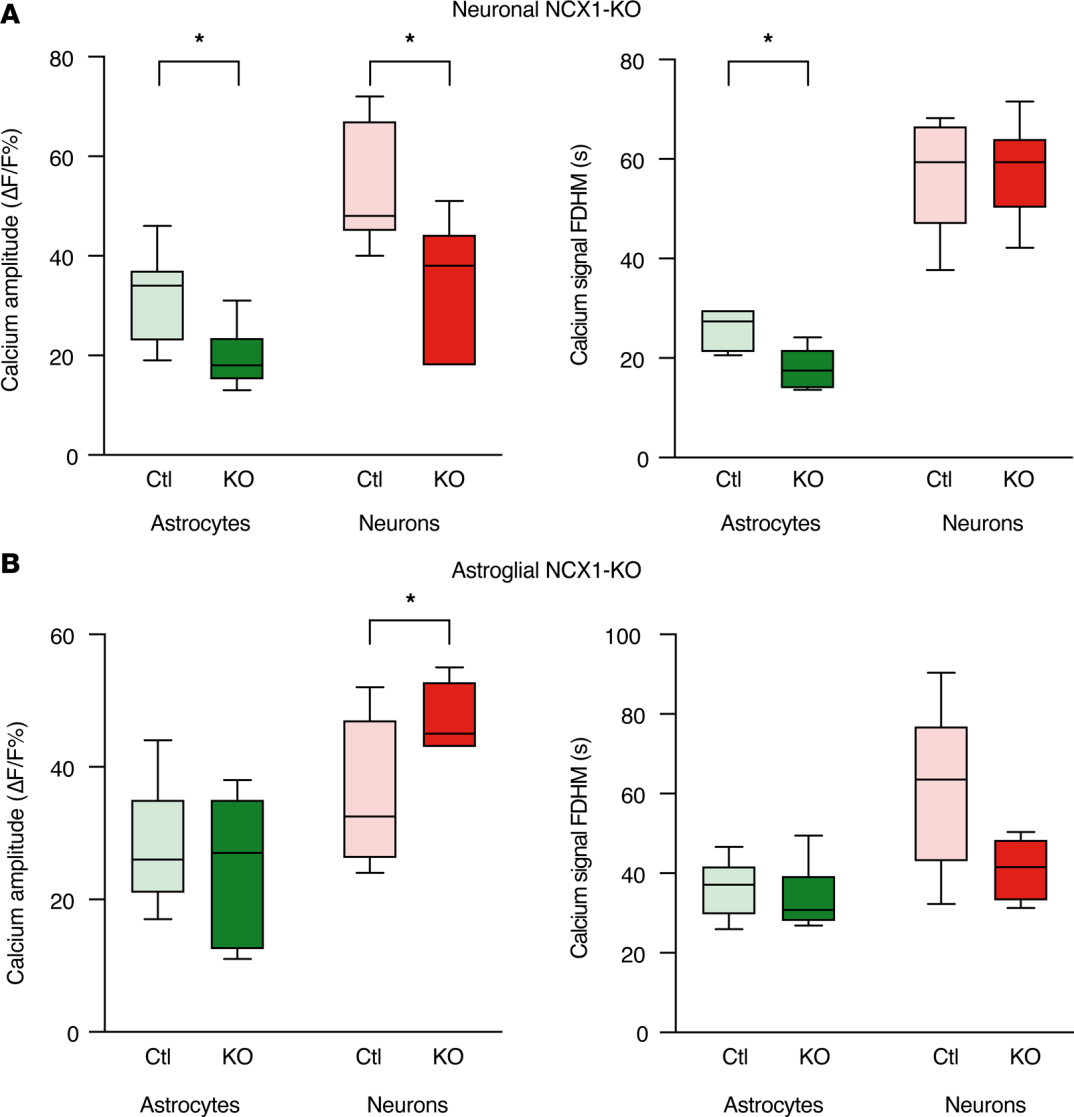
Neuronal and astroglial NCX1 deletion differentially modulate calcium changes during ischemia-induced spreading depolarizations. (**A**) Relative calcium changes (ΔF/F%) and durations (full duration at half-maximum, FDHM) in neuronal NCX1-KO and Cre-negative control mice were determined in astrocytes and neurons. Calcium amplitudes were significantly higher in astrocytes and neurons, and significantly longer in astrocytes (*P < 0.05; Mann-Whitney test for all comparisons; *n* = 6 KO and *n* = 7 control animals). (**B**) ΔF/F% and FDHM in astroglial NCX1-KO and Cre-negative control mice were determined in astrocytes and neurons. Calcium amplitudes were significantly higher in astrocytes (*P < 0.05; Mann-Whitney test for all comparisons; *n* = 6 KO and *n* = 8 control animals).

Comparing neuronal NCX1-KO mice to Cre-negative littermate controls, we found that peak amplitudes of spreading depolarization–related calcium elevations were lower in neurons as well as in astrocytes compared with neurons and astrocytes in control mice, and that their duration was shorter in astrocytes (Figure 2A). In contrast, when we deleted NCX1 specifically in astrocytes, this resulted in significantly higher calcium amplitudes in neurons during spreading depolarizations, whereas calcium changes in astrocytes remained unchanged (Figure 2B).

### Neuronal, but not astroglial, NCX1 contributes to spreading depolarization threshold after stroke.

We next analyzed whether cell-selective deletion of NCX1 altered the propensity of ischemic cortex to spreading depolarizations. To this end, we measured the delay to the first occurrence of the stereotypical propagating calcium wavefront in astrocytes and neurons associated with spreading depolarizations (23) as well as their frequency within the first 90 minutes after ischemia induction, defined by the initiation of MCAO.

**Figure 3:**
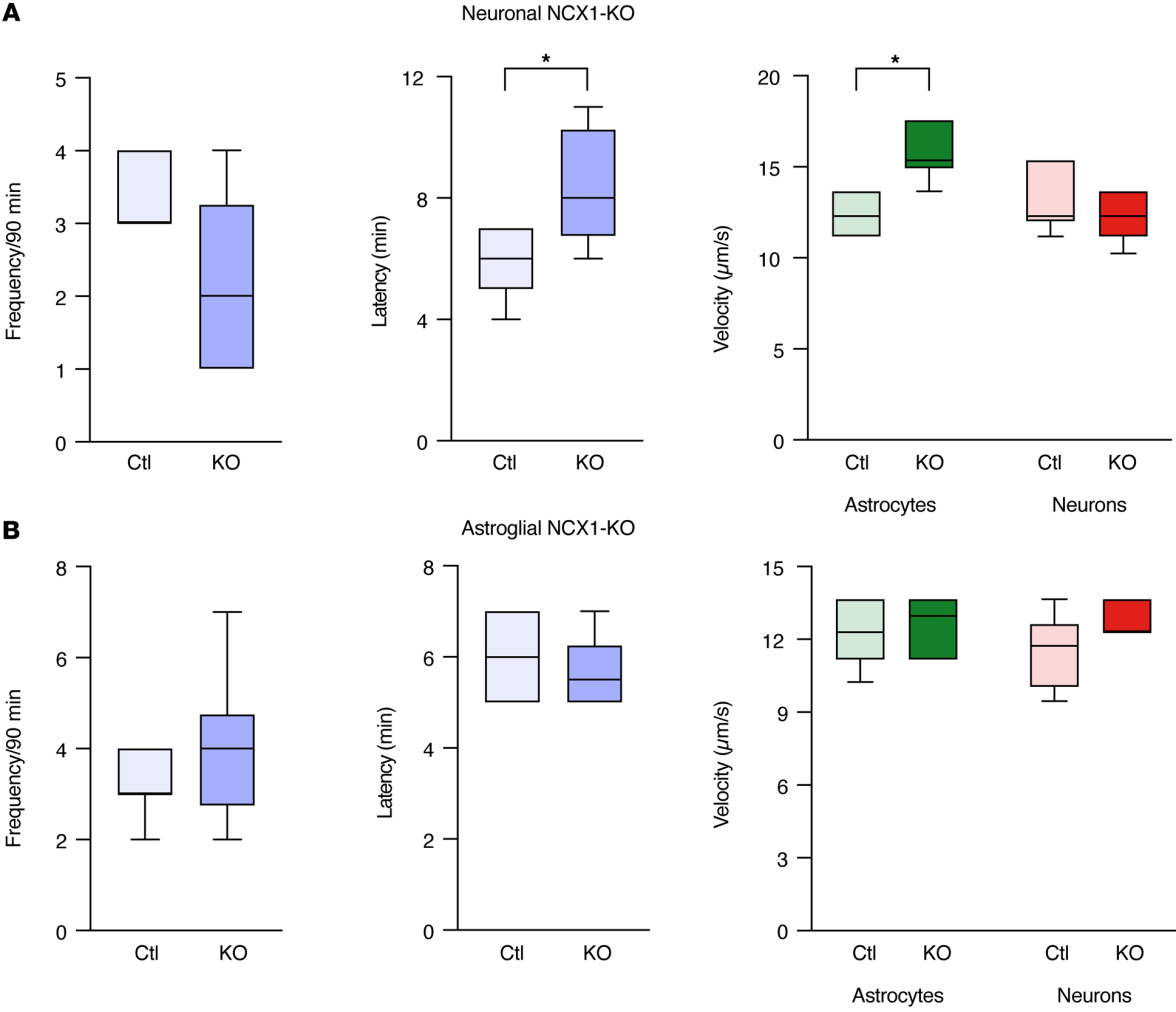
Neuronal, but not astroglial, NCX1 deletion modulates spreading depolarization threshold during ischemia. (**A**) Spreading depolarization threshold and cell-specific velocity in neuronal NCX1-KO and Cre-negative control mice. Spreading depolarization frequency in the first 90 minutes after MCAO induction was non-significantly reduced, and the latency to the first spreading depolarization was significantly higher in KO mice. The velocity of the calcium wave accompanying spreading depolarizations was significantly higher in astrocytes, but remained unchanged in neurons (*P < 0.05; Mann-Whitney test for all comparisons; *n* = 6 KO and *n* = 7 control animals). (**B**) Spreading depolarization frequency and latency, as well as the calcium wave velocities were similar in astroglial NCX1-KO mice compared to Cre-negative control mice (*P < 0.05; Mann-Whitney test for all comparisons; *n* = 6 KO and *n* = 7 control animals).

Interestingly, we found that neuronal NCX1 deletion resulted in a higher spreading depolarization threshold, indicated by a significantly longer latency to the appearance of the first spreading depolarization after the induction of ischemia and a nonsignificant trend to lower spreading depolarization frequency (Figure 3A). We also found that the velocity of the spreading depolarization–associated astroglial calcium wave was higher in astrocytes, but unchanged in neurons (Figure 3A). In turn, astrocyte-specific deletion of NCX1 did not cause significant changes in any of these parameters (Figure 3B).

### Differential regulation of spreading depolarization–associated sodium changes after neuronal and astroglial NCX1 deletion.

**Figure 4:**
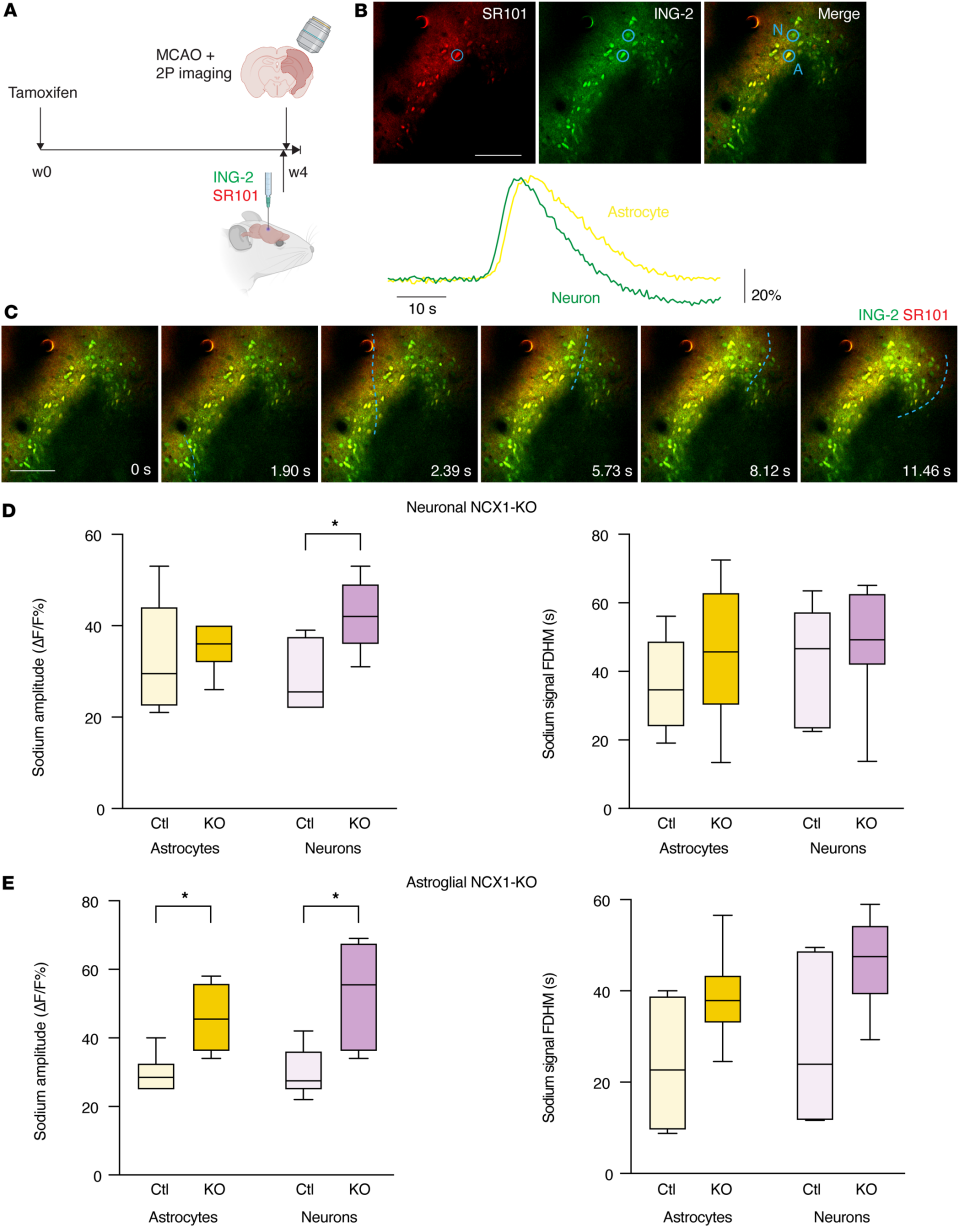
Neuronal and astroglial NCX1 deletion differentially modulate sodium changes during ischemia-induced spreading depolarizations. (**A**) Experimental paradigm. Four weeks after Cre activation using tamoxifen, the sodium indicator ING-2 and the astrocyte reporter SR101 were co-injected into cortex, and mice were subsequently subjected to MCAO and imaged. (**B** and **C**) Ischemia-induced spreading depolarizations were accompanied by transient propagating elevations in sodium in neurons and astrocytes (the latter were identified by their colabeling with SR101 and appear yellow in merged images). Scale bars: 100 μm. (**D**) In neuron-specific NCX1-KO mice, the relative sodium amplitude (ΔF/F%) was significantly higher in neurons, but not in astrocytes, and the duration of sodium transients (FDHM) was similar (*P < 0.05; Mann-Whitney test for all comparisons; *n* = 7 KO and *n* = 6 control animals). (**E**) In astrocyte-specific NCX1-KO mice, sodium amplitudes were significantly higher in astrocytes and neurons in KO mice, while the duration of sodium transients was similar (*P < 0.05; Mann-Whitney test for all comparisons; *n* = 6 KO and *n* = 6 control animals).

As NCX1 functions as a Na^+^/Ca^2+^ antiporter, we next investigated whether the cell-specific modulation of calcium transients would also be accompanied by reciprocal changes in sodium. To this end, we injected the sodium indicator ING-2 AM (24) together with the astrocyte-specific dye sulforhodamine 101 (SR101) (25) into the cortex shortly before MCAO (Figure 4A). As reported previously (16), cortical spreading depolarizations during ischemia were accompanied by strong propagating and transient increases in sodium in neurons and astrocytes (identified by their colabeling with SR101; Figure 4, B and C).

In neuron-specific NCX1-KO mice, the sodium amplitude was significantly higher in neurons (Figure 4D), compatible with the role of NCX1 as a (reverse) sodium-calcium exchanger and the lower calcium amplitudes observed during spreading depolarizations in these mice (Figure 2A).

Given that spreading depolarization–associated calcium amplitudes were higher in astrocyte-specific NCX1-KO mice (Figure 2B), we expected lower spreading depolarization–associated sodium amplitudes in these mice. Surprisingly, however, we observed that sodium amplitudes were higher in astrocytes as well as neurons in astrocyte-specific NCX1-KO mice compared with controls (Figure 4E).

### Spreading depolarization–evoked glutamate levels are lower in neuron-specific NCX1-KO but higher in astrocyte-specific NCX1-KO mice.

**Figure 5:**
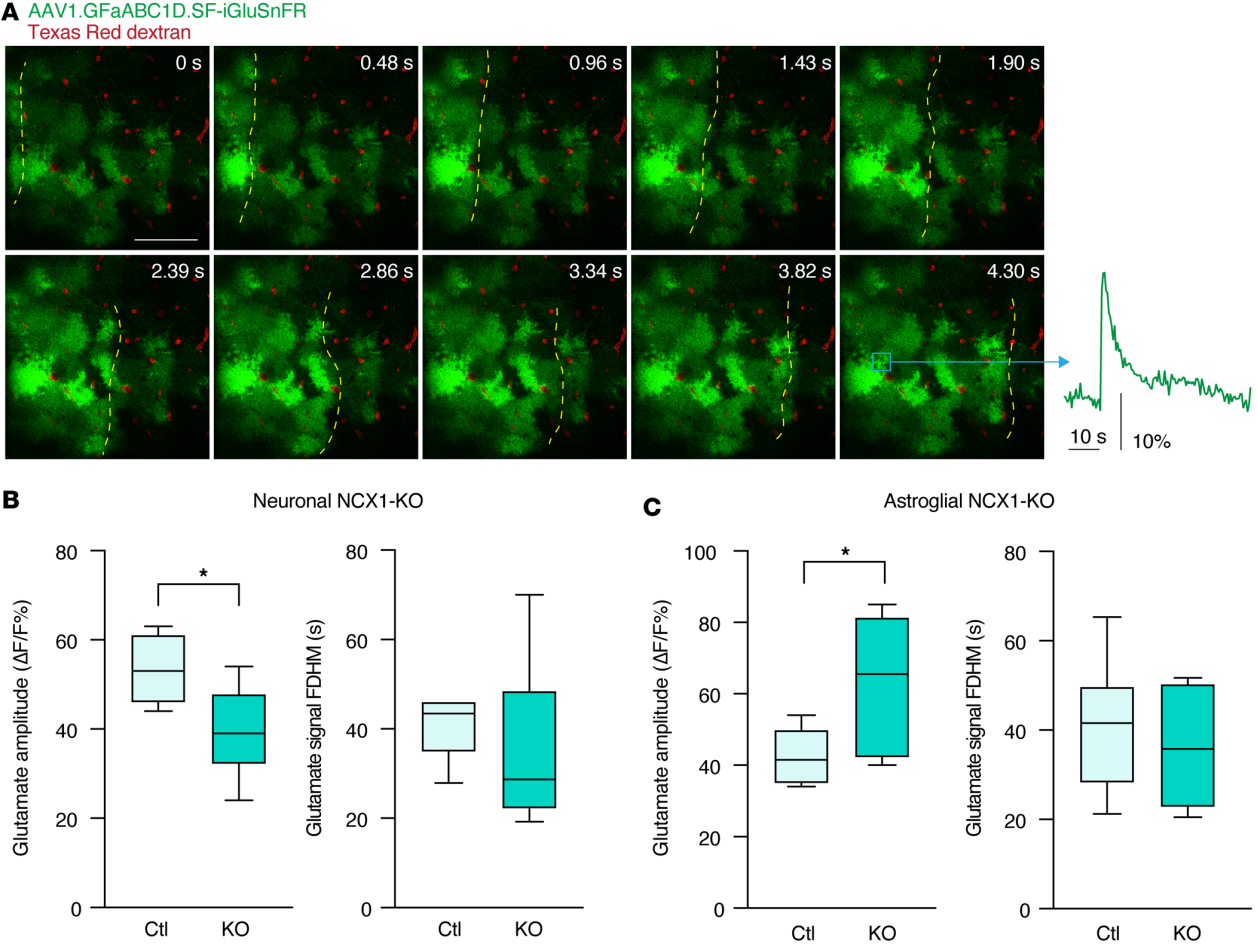
Opposite effects of cell-selective NCX1 deletion on extracellular glutamate levels. (**A**) The glutamate reporter AAV1.GFAP.SF-iGluSnFr was injected into cortex to transduce astrocytes and measure extracellular glutamate levels; spreading depolarizations after ischemia were associated with strong extracellular glutamate transients. Scale bar: 100 μm. (**B**) In neuron-specific NCX1-KO mice, spreading depolarization–induced extracellular glutamate amplitudes were significantly lower compared with Cre-negative control mice; the duration was similar (*P < 0.05; Mann-Whitney test for all comparisons; *n* = 8 KO and *n* = 7 control animals). (**C**) In astrocyte-specific NCX1-KO mice, spreading depolarization–induced extracellular glutamate amplitudes were significantly higher compared with control mice; the duration was similar (*P < 0.05; Mann-Whitney test for all comparisons; *n* = 6 KO and *n* = 8 control animals).

Given the consequences of calcium and sodium elevations during spreading depolarization for glutamate release (8, 26), we next investigated extrasynaptic extracellular glutamate levels in both KO models. To this end, we injected the glutamate sensor AAV1.GfaABC1D.SF-iGluSnFR (27) into the cortex 1 week prior to tamoxifen administration. This resulted in labeling of astrocytes with the reporter (Figure 5A). As reported previously (8), spreading depolarizations after ischemia induced strong extracellular glutamate transients (Figure 5A).

Given the strong dependence of spreading depolarization–associated glutamate release on intracellular calcium (8), we expected that the amplitude of extracellular glutamate levels during spreading depolarizations would follow the direction of calcium changes in both cell-specific KO models. Indeed, we found that the spreading depolarization–induced extracellular glutamate amplitude was lower in neuron-specific NCX1-KO mice (Figure 5B), in line with smaller calcium amplitudes in these mice described above (Figure 2A). In contrast, glutamate amplitudes were higher in astrocyte-specific NCX1-KO mice (Figure 5C), compatible with the higher calcium amplitudes observed in these mice (Figure 2B).

### Modeling the effect of astroglial NCX1 deletion on calcium, sodium, and glutamate.

To summarize the data obtained in neuronal NCX1-KO mice, we found that the specific deletion of NCX1 in neurons led to smaller calcium transients in astrocytes and neurons, stronger neuronal sodium transients, lower extracellular glutamate elevations, and a higher threshold for spreading depolarizations. These changes are explainable by reverse operation of neuronal NCX1 — i.e., calcium import into cells in exchange for sodium — so that neuronal NCX1 deletion leads to higher sodium and lower calcium levels in neurons. The lower calcium levels in neurons reduce synaptic glutamate release and increase spreading depolarization threshold. In turn, this lower extracellular glutamate leads to lower calcium elevations in astrocytes.

On the other hand, the changes observed in astrocyte-specific NCX1-KO mice were more complex. Specifically, we found that intracellular sodium amplitudes were higher in astrocytes and neurons, and that calcium amplitudes were unchanged in astrocytes and higher in neurons in astrocyte-specific NCX1-KO mice during spreading depolarizations. This is not explainable on the basis of NCX1 activity alone, since deleting astroglial reverse-mode NCX1 should decrease intracellular calcium in astrocytes. We also observed higher extracellular glutamate levels in astrocyte-specific NCX1-KO mice during spreading depolarizations. We therefore reasoned that in these mice, increased intracellular sodium levels in astrocytes due to NCX1 KO (considering the operation of NCX1 in reverse mode) would lead to a reversal (or at least a strong reduction in the activity) of the astroglial glutamate transporter GLT1 (EAAT2) (28). This would result in the observed increase in extracellular glutamate levels, which in turn would activate astroglial glutamate receptors, such as metabotropic glutamate receptors (mGluRs), compensating for the decreased calcium levels induced by NCX1 deletion in these mice. Higher extracellular glutamate levels (due to the reversal of EAAT2) would then lead to higher neuronal calcium and sodium by stimulating neuronal glutamate receptors such as NMDA and AMPA.

**Figure 6:**
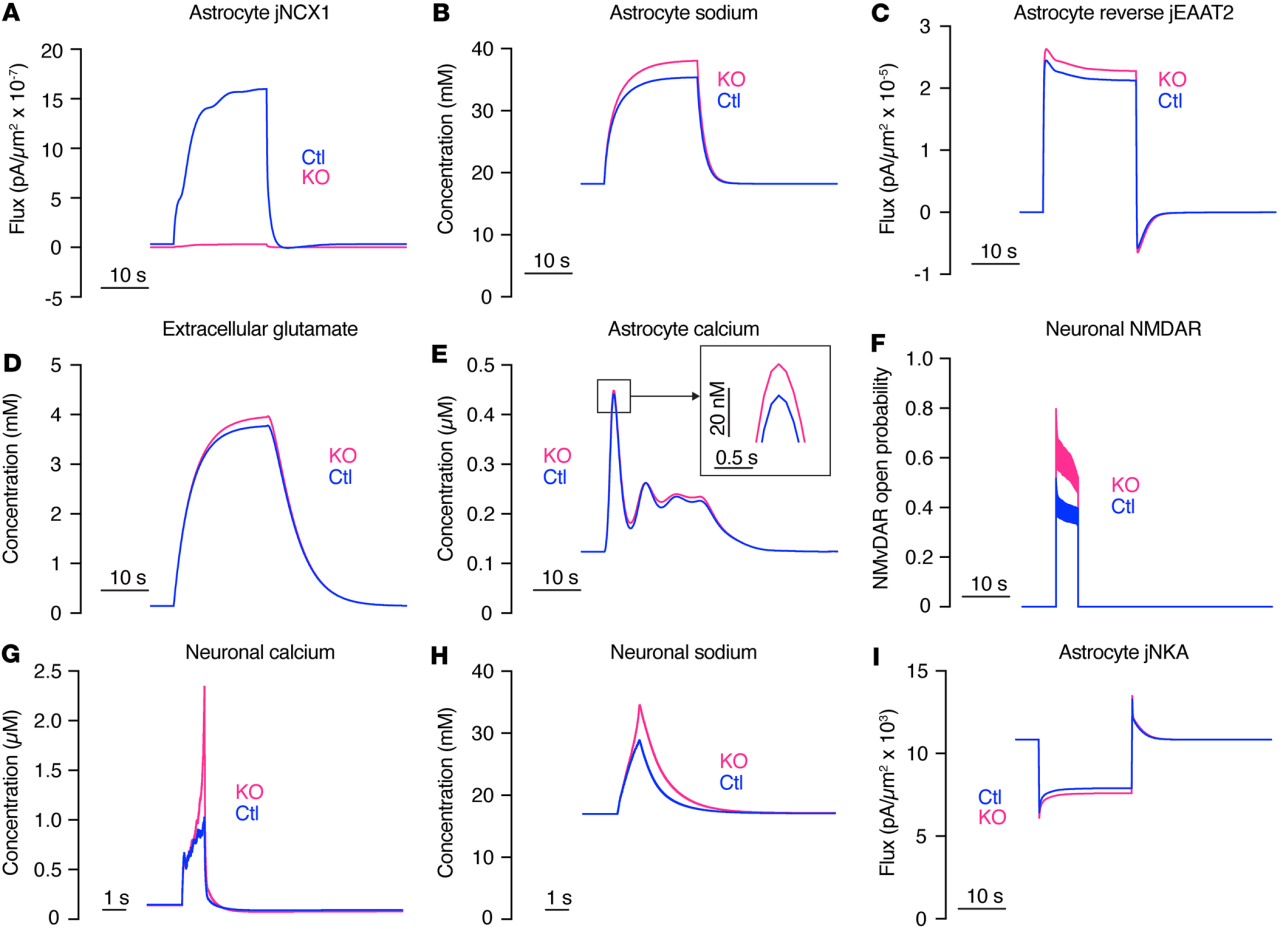
Computational modeling supports reverse-mode NCX1 action in astrocyte-specific NCX1-KO mice during spreading depolarization under simulated ischemia. In this model, energy deprivation during ischemia and spreading depolarization was simulated by reducing the activity of the Na^+^/K^+^-ATPase to 50% for 20 seconds. Astrocyte-specific NCX1 deletion was modeled by a 99% reduction in NCX1 activity in astrocytes. (**A**) Astrocyte-specific NCX1 deletion was modeled by a 99% reduction in NCX1 activity in astrocytes (expressed as NCX1 flux, jNCX1). (**B**) Spreading depolarization under simulated ischemia is associated with higher astroglial peak sodium under KO conditions compared with control. (**C**) Flux through reverse-mode glutamate transporter GLT1 (jEAAT2) increases due to astroglial NCX1 KO (positive and negative values along *y*-axis indicate reverse and forward mode, respectively). (**D** and **E**) Higher reverse-mode EAAT2 flux under KO conditions leads to a higher glutamate concentration in the extracellular space, which overcompensates the drop in calcium due to NCX1 deletion, resulting in a slight intracellular calcium increase. (**F**) This higher extracellular glutamate results in higher activity of neuronal NMDA receptors (NMDARs, expressed as open probability). (**G** and **H**) In neurons, higher NMDAR activity leads to higher peak sodium and calcium levels as compared with control. (**I**) Na^+^/K^+^-ATPase activity (expressed as flux, jNKA) during ischemic spreading depolarization is higher in NCX1-KO astrocytes.

To test this hypothesis, we developed a detailed biophysical model replicating key observations about sodium and calcium changes in cortical neurons and astrocytes during ischemia and spreading depolarization (see Methods) (29, 30). In this model, energy loss during ischemia and spreading depolarization was simulated by reducing the activity of the Na^+^/K^+^-ATPase to 50% of its normal value for 20 seconds. Astrocyte-specific NCX1 deletion was modeled by a 99% reduction in NCX1 activity in astrocytes (Figure 6A).

Consistent with our observations, the model shows that ischemia leads to a higher astroglial peak sodium in astrocyte-specific NCX1-KO conditions compared with control (Figure 6B). As hypothesized, this leads to an increased flux through EAAT2 in the reverse mode under astroglial NCX1-KO conditions (Figure 6C). This higher reverse-mode EAAT2 flux leads to a higher glutamate concentration in the extracellular space (Figure 6D), which in turn results in a larger calcium release from intracellular stores in the astrocyte through mGluR-mediated pathways, overcompensating the drop in calcium due to NCX1 deletion (Figure 6E).

To investigate the effect of astrocyte-specific NCX1 deletion on neuronal ion homeostasis, we exposed the model neuron to the same extracellular conditions as in Figure 6, A–E, including simulated ischemia by a reduction of Na^+^/K^+^-ATPase peak capacity to 50%. We found that the higher extracellular glutamate concentration (Figure 6D), due to higher reverse-mode EAAT2 flux, leads to a stronger activation of neuronal NMDA receptors (Figure 6F). This, in turn, leads to higher peak calcium (Figure 6G) and sodium levels (Figure 6H) in the neuron under astrocyte-specific NCX1-KO as compared with control conditions. We also noted that the impairment of Na^+^/K^+^-ATPase activity due to ischemia is slightly higher in NCX1-KO astrocytes (Figure 6I), which may also contribute to the higher intracellular sodium compared with control astrocytes (Figure 6B).

## Discussion

Here, we investigated the roles of NCX in neurons and astrocytes during spreading depolarizations after ischemia. We found that neuronal deletion of NCX1 resulted in smaller calcium changes in both cell types, higher intracellular sodium transients, and lower extracellular glutamate transients, altogether resulting in a higher threshold for spreading depolarizations. On the other hand, NCX1 deletion selectively in astrocytes led to sodium changes that were higher both in neurons and astrocytes compared with controls, as well as higher calcium changes in neurons and higher extracellular glutamate levels.

**Figure 7:**
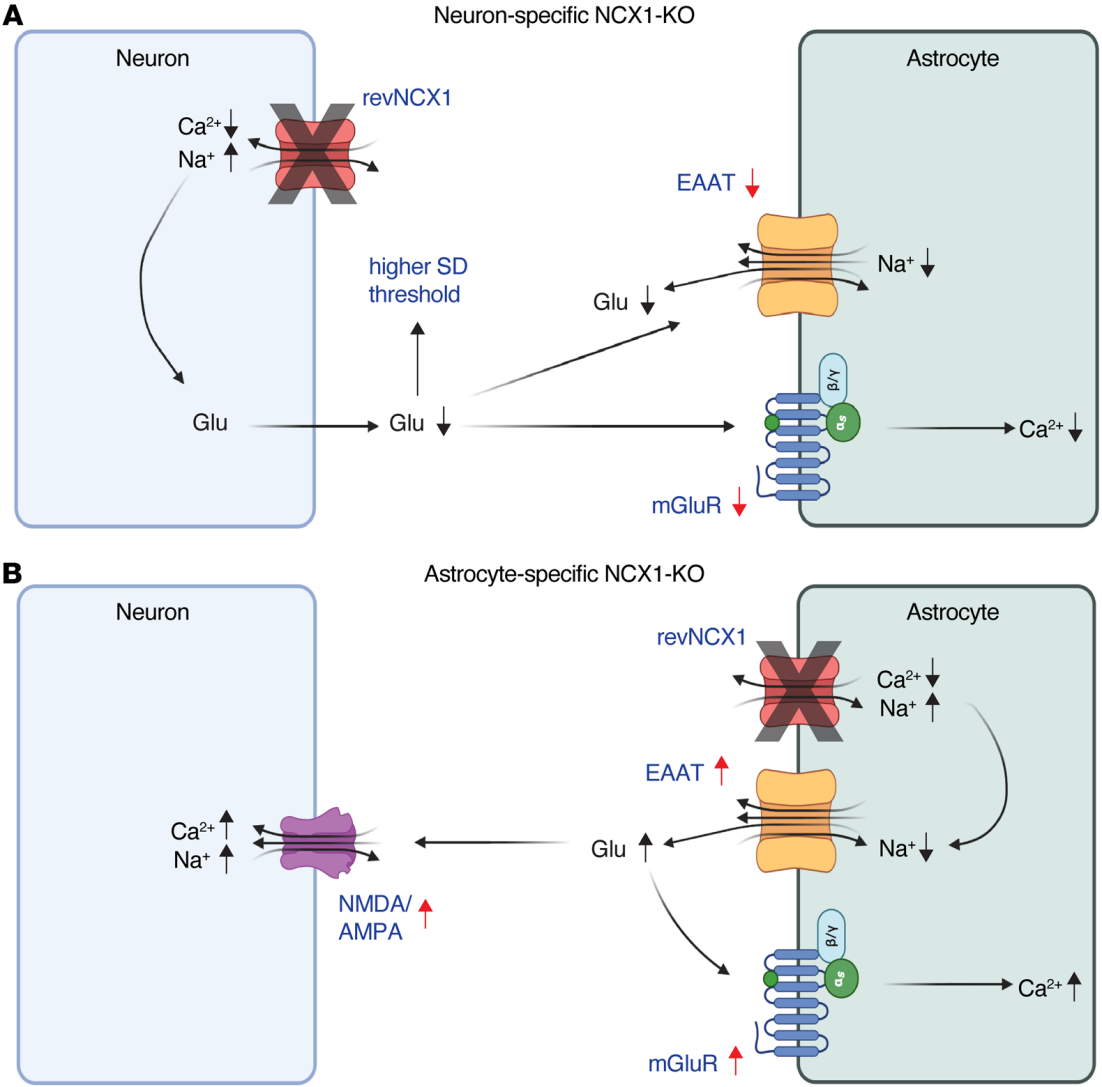
Summary of proposed ion and glutamate changes in cell-selective NCX1-KO mice. (**A**) In neuron-specific NCX KO mice, deleting reverse-mode NCX1 leads to higher sodium and lower calcium levels, which in turn reduces neuronal glutamate release, resulting in a higher spreading depolarization (SD) threshold. This reduces the driving force for reverse astroglial glutamate transporter (EAAT/GLT1) as well as metabotropic glutamate receptor (mGluR) activation on astrocytes, together reducing calcium and slightly increasing sodium levels. (**B**) In astrocyte-specific NCX-KO mice, deleting reverse-mode NCX1 leads to higher sodium and lower calcium levels, which increases glutamate release through reverse EAAT. This, in turn, activates astroglial mGluR, leading to a compensation of calcium levels in astrocytes, and induces higher calcium and sodium levels in neurons through NMDA and AMPA receptor activation.

We propose that the data on neuron-specific NCX KO are explainable by NCX functioning in the reverse mode. That is, intracellular sodium levels increase as a result of ATP shortage and subsequent Na^+^/K^+^-ATPase dysfunction (12, 13), leading to export of sodium and import of calcium into neurons through NCX. The resulting calcium overload results in an amplified glutamate release from neurons, subsequently leading to higher calcium in astrocytes and a lower threshold for spreading depolarizations (Figure 7A). Hence, neuronal reverse-mode NCX contributes to the detrimental calcium overload and spreading depolarization development after ischemia. Interestingly, we also observed a higher velocity of the spreading depolarization–associated astroglial calcium wave in astrocytes in neuron-specific NCX-KO mice, perhaps indicating that stronger calcium propagation between astrocytes may protect against spreading depolarization as suggested by previous studies (31).

On the other hand, the role of NCX in astrocytes seemed more complex, in that calcium and sodium changes were altered in the same direction. Gleaning from a detailed computational model that incorporates key biophysical data in cortical neurons and astrocytes during ischemia and spreading depolarizations (29, 30), we propose that, perhaps counterintuitively, these changes are also explainable by astroglial NCX functioning in the reverse mode. Specifically, our model predicts that, as expected, a deletion of reverse-mode NCX in astrocytes leads to an increase in sodium in astrocytes. As our model predicts that the astroglial glutamate transporter GLT1 (EAAT2) predominantly operates in reverse mode during spreading depolarization, this increased sodium load induced by NCX deletion would further increase the reverse flux through EAAT2, leading to the observed higher extracellular glutamate levels in astrocyte-specific NCX-KO mice. The increased glutamate, in turn, leads to a stronger activation of astroglial glutamate receptors, such as mGluRs, leveling out — in fact, slightly overcompensating in our model — the decreased calcium levels induced by reverse-mode NCX1 deletion in astrocytes through calcium release from intracellular stores. This glutamate increase, in turn, also induces higher peak calcium and sodium levels in neurons during spreading depolarizations through NMDA receptor activation (Figure 7B).

Extrapolating these experimental and computational data to pathophysiological conditions, we propose that reverse-mode NCX in astrocytes during spreading depolarizations after ischemia has no major effect on astroglial calcium elevations. Reverse NCX in astrocytes, however, augments the forward activity of GLT1 due to the export of sodium in astrocytes, leading to lower extracellular glutamate levels and lower NMDA receptor–dependent calcium and sodium influx into neurons.

On the other hand, our data imply that the effects of NCX on spreading depolarization are exclusively driven by neuronal NCX. The net effect of the observed and modeled changes of reverse-mode NCX during spreading depolarizations are that the actions of reverse-mode NCX in neurons would be detrimental to neuronal survival, and that those in astrocytes would be protective. Interestingly, previous studies have shown that genetic deletion or pharmacological inhibition of NCX can either have detrimental (32–35) or beneficial effects (14, 15, 36) in models of ischemic brain injury, indicating that these seemingly contradictory results may in fact reflect opposing roles of astrocytes and neurons. However, we here specifically investigated the role of NCX in spreading depolarizations in penumbral cortex. The directional mode of NCX in different cell types and the consequences for ion fluxes and neuronal survival during, but also outside of, spreading depolarizations likely depend on the level of metabolic compromise, and need to be investigated in future studies. Moreover, whether outcome is largely dependent on calcium, or if and how sodium changes can also modulate neuronal survival during ischemia in neurons and astrocytes, remains to be determined in future studies.

In summary, our work implies that neuronal reverse-mode NCX contributes to the detrimental calcium overload and spreading depolarization development after ischemia, while reverse-mode NCX in astrocytes during ischemic spreading depolarizations is protective by lowering extracellular glutamate. These diverse roles will have to be taken into account when considering NCX as a potential translational target in stroke.

## Methods

### Sex as a biological variable.

Equal numbers of male and female mice were used, and no significant differences were found between both sexes.

### Animals.

Experiments were performed in 3- to 5-month-old mice. All mice were on a C57BL/6 background. Hemizygous *Camk2a*-CreERT2 mice [Tg(*Camk2a*-cre/ERT2)1Aibs/J; Jackson Laboratory, strain 012362] (19) or hemizygous *Glast*-CreERT mice [Tg(*Slc1a3*-cre/ERT)1Nat/J; Jackson Laboratory, strain 012586] (20) were crossed with NCX1-loxP mice (*Slc8a1*^tm1Kdp^/J; Jackson Laboratory, strain 025943), in which exon 11 of the *Slc8a1* gene is flanked by loxP sites (21). Cre recombination was induced by tamoxifen injections (5 μL/g; Sigma-Aldrich; solubilized in ethanol with sunflower oil) i.p. for 5 days. Animals were housed in groups on a 12-hour light/dark cycle with food and water available ad libitum.

### Cranial window preparations and intracortical injections.

Chronic cranial windows were prepared as described previously (37). Briefly, animals received buprenorphine as an analgesic (0.1 mg/kg), dexamethasone as an antiinflammatory drug (0.2 mg/kg), and cefotaxime as an antibiotic (2 g/kg), were anesthetized with isoflurane (induction, 3%; maintenance, 1%–1.5% vol/vol) and kept on a heating pad (37°C; World Precision Instruments [WPI]). After fixation in a stereotactic frame, the scalp was removed, and a craniotomy (diameter, 3 mm) was performed above the left somatosensory cortex (coordinates: –2.0 mm posterior and +1.5 mm lateral from bregma) using a dental drill. After dura removal, 1 μL of adeno-associated virus (AAV) was injected (AAV1.Syn.NES-jRGECO1a.WPRE.SV40, 100854-AAV1; AAV5-GFaABV1D-cyto-GCaMP6f, 52925-AAV5; AAV1.GFAP.SF-iGluSnFr.A184S, 106192-AAV1; Addgene) at 0.1 μL/min into the cortex at a depth of 100–150 μm using a glass micropipette (6 μm tip diameter; WPI) connected to a Hamilton syringe attached to a pump (MICRO4, Ultra Microinjection Syringe Pump, WPI). The glass pipette was left in place for 2 minutes before removal. The window was closed with a cover glass (diameter, 4 mm) and sealed using UV-cured flowable composite (Gradia DirectFlo, GC Corporation). Mice were allowed to recover in a warmed recovery chamber (V1200, Peco) and scanned 4–5 weeks later.

Acute cranial windows were prepared as described above, with the difference that the indicator Natrium Green-2 acetoxymethyl ester (ING-2 AM, 2011F, ION Biosciences; solubilized 1:10 in 20% Pluronic and 80% dimethyl sulfoxide [DMSO; Sigma-Aldrich]) was co-injected together with the astrocytic marker sulforhodamine-101 (100 μM; Sigma-Aldrich) into cortex (total injection volume, 2 μL) at a depth of 100–150 μm using glass micropipettes (4–6 μm tip diameter; WPI) connected to a pneumatic injector (PDES, NPI Electronic). Agarose (1.5%) was placed on top of the cortex for stabilization, and the window was closed with a cover glass and sealed with dental cement.

### Focal cerebral ischemia.

Ischemia was induced by occlusion of the left middle cerebral artery (MCAO), as described previously (8, 26, 38, 39). Briefly, mice were administered buprenorphine s.c. (0.1 mg/kg) and anesthetized with isoflurane (induction, 3%; maintenance, 1%–1.5% vol/vol). Body temperature was controlled during surgery via a temperature-regulated heating pad. The left common carotid artery (CCA), the external carotid artery, and the internal carotid artery (ICA) were exposed. A 7-0 silicone rubber coated monofilament with a coating length of 9–10 mm, a total length of 20 mm, and a tip diameter of 0.19 ± 0.01 mm (7019910PK5Re, Doccol) was introduced through a small incision in the CCA and inserted further into the ICA and circle of Willis to occlude the entrance to the middle cerebral artery, and fixed in place with a suture.

### Multiphoton microscopy.

During imaging, mice were anesthetized with 1%–1.5% isoflurane on a homeothermic heating pad. To visualize the vasculature, dextran-coupled Texas Red (70 kDa, 5% in saline, 30 μL; Invitrogen) or Cascade Blue dextran (10 kDa, 5% in saline, 30 μL; D1976, Invitrogen) were injected intravenously. Mice were imaged through the cranial windows using an upright 2-photon microscope (LaVision Trim ScopeII with optical parameter oscillator) with a 20× objective (1.0 numerical aperture, Zeiss) and 3 non-descanned detectors with bandpass filters (460–80, 525–50, and 617–73 nm) at 350 × 350 pixels (0.69 μm/pixel). Fluorophores were excited at 830 nm (ING-2, Cascade Blue dextran), 920 nm (SF-iGluSnFR), and 1100 nm (jRGECO1a) using a Ti:Sapphire laser (Chameleon Ultra II, pumped by an 18 W laser; Coherent). To minimize phototoxicity and laser-induced artifacts, laser power below the objective was kept at 20–40 mW. *Z*-stacks of the imaging region were acquired (364 × 364, 1024 px, pixel size 0.36 μm, 0.65 μs). For dynamic imaging, XY time-lapse series (350 × 350, 512 px, pixel size 0.69 μm) at 2.09 Hz were recorded for 12 minutes at a depth of 100–150 μm beneath the pial surface.

### Immunohistochemistry.

Mice were transcardially perfused under deep anesthesia/analgesia (isoflurane, 3%; ketamine, 1 mg/kg i.p.) with phosphate-buffered saline (PBS). Subsequently, brains were fixed in 4% paraformaldehyde overnight, and then cryoprotected by immersion in sucrose (15% and 25%), and embedded in Tissue-Tek (Sakura). Coronal sections (20 μm) were cut using a cryostat (Leica Microsystems), mounted, and stored at –20°C. For immunostainings, cryosections from the infarct area were kept at room temperature for 30 minutes, preincubated with 10% normal serum (Vector Labs) and 0.3% Triton X-100 (Sigma-Aldrich) in PBS for 1 hour, and then incubated overnight at 4°C with the following primary antibodies: rat anti-GFAP (1:500; 13-0300, Invitrogen), rabbit anti-NCX1 (1:200; BS-1550R, Thermo Fisher Scientific), and mouse anti-NeuN (1:50; MAB377, Millipore) in 5% normal goat serum and 0.05% Triton X-100. For nuclear staining, DAPI (1:1000; Thermo Fisher Scientific) was added to the sections with secondary antibodies (all from Invitrogen). Confocal images were acquired using a confocal laser-scanning microscope (Zeiss LSM 900).

### Modeling ion homeostasis in the astrocyte, neuron, and extracellular space.

A schematic of the key pathways included in the model is shown in Supplemental Figure 1; supplemental material available online with this article; https://doi.org/10.1172/jci.insight.200411DS1 For the astrocyte, we extended our previous model of intracellular sodium ([Na^+^]_ai_), potassium ([K^+^]_ai_), chloride ([Cl^−^]_ai_), and calcium ([Ca^2+^]_ai_) concentrations (29, 40, 41) by incorporating glutamate homeostasis. We also modeled ion homeostasis in the neuron. Since the glutamate transporter (EAAT2) cotransports 3 Na^+^ and 1 H^+^ and countertransports 1 K^+^ for each glutamate molecule, the equations for intra- and extracellular Na^+^, K^+^, and membrane potential are changed accordingly. Various equations used in the model for the astrocyte are given in the supplemental material. Here we present the modifications made to the previous model.

### Extracellular glutamate dynamics.

The dynamics of extracellular glutamate concentration ([glu]_o_) were modeled according to Equation 1, as in Passlick et al. (42).

 
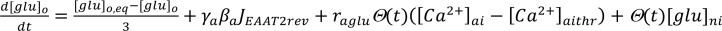
Equation 1

The term [*glu*]*_o,eq_* represents the concentration of [glu]_o_ under resting conditions. Parameters β_α_ represents the volume ratio of astrocyte to extracellular space and γ_α_ converts current (pA/μm^2^) to flux (μM/sec) (29). We assume that under our experimental ischemic conditions, EAAT2 operates in reverse mode as described in Rossi et al. (43), represented by *J_EAAT2rev_*, which is modeled as Equation 2.


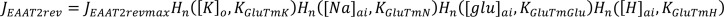
Equation 2

*J_EAAT2max_* is the maximum current through glutamate transporters. *H*(*n*, *X*, *K*) is the Hill function of the form *X^n^*/(*K^n^* + *X^n^*), where *n*, *X*, and *K* represent the number of ions, ion concentration (i.e., Na^+^, K^+^, Glu, and H^+^), and their corresponding binding affinities for the transporter, respectively. The values of intracellular glutamate ([glu]_ai_) and proton ([H^+^]_ai_) concentration were calibrated to represent the conditions under ischemic stroke, as described in Rossi et al. (43). That is, [glu]_ai_ was assumed to decrease from 5 mM to 3 mM and [H^+^]_ai_ was set to 141 nM under ischemic stroke (43).

Glutamate release by the neuron ([glu]_ni_) was modeled by raising extracellular glutamate by 1 mM for 20 seconds using a Heaviside step function, Θ(t), as in Thapaliya et al. (29). Our model also incorporates Ca^2+^-dependent glutamate release by astrocytes with a peak release rate *r_aglu_* (second-last term in Equation 1). This release occurs when [Ca^2+^]_ai_ exceeds a critical threshold, [Ca^2+^]_aithr_.

### Modeling neuronal ion homeostasis.

To understand the effect of astrocytic NCX1 KO on neurons during ischemia, we modified the model from Wei et al. (44) by incorporating the fluxes through NCX1 transporter, NMDA, and AMPA receptors, as shown in Supplemental Figure 1. The neuronal Na^+^ concentration ([Na^+^]_ni_) is regulated by voltage-gated Na^+^ channels (*J_nNa_*), Na^+^/K^+^-ATPase (*J_nNaK_*), NMDA receptors (*J_nNMDA_*), AMPA receptors (*J_nAMPA_*), Na^+^/K^+^/Cl^–^ cotransporter 1 (*J_nkcc1_*), and NCX1 (*J_nNCX_*). Accordingly, the rate of change in [Na^+^]_ni_ is provided by Equation 3.


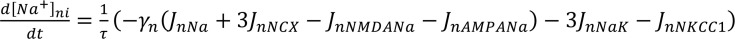
Equation 3

The term *τ* converts seconds to milliseconds and *γ_n_* = *S*/(*F* × *v_ni_*) is a conversion factor from current (A/cm^2^) to flux (mM/s). *S*, *v_ni_*, and *F* are the surface area of the cell, intracellular volume, and Faraday constant, respectively. Equation 4 was used for the fluxes due to neuronal voltage-gated and leak Na^+^ channels (44).


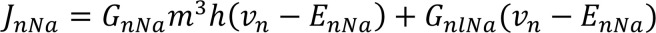
Equation 4

The terms *G_nNa_, G_nlNa_, v_n_,* and *E_nNa_* are the peak conductance due to neuronal voltage-gated channels, Na^+^ leak, membrane potential, and reversal potential for Na^+^, respectively. The activation and inactivation variables *m* and *h* are described in the supplemental material.

Equation 5 was used for the flux due to Na^+^/K^+^-ATPase (44).


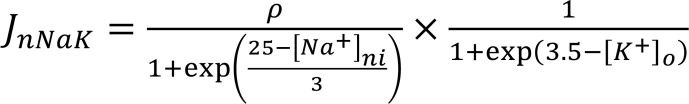
Equation 5

The term *ρ* represents the pump strength, and depends on the available oxygen (Equation 6).

,
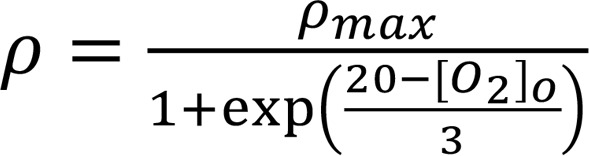
Equation 6

and *p_max_* is the maximum pump strength at normal oxygen levels.

For simplicity, we used the same expression for the neuronal NCX flux *J_nNCX_* as described for the astrocyte, substituting the astrocytic ion concentrations with neuronal ion concentrations.

The fluxes due to neuronal AMPA and NMDA receptors are modeled in Equations 7 and 8, as in Rossi et al. (43).


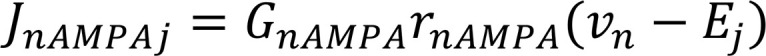
Equation 7


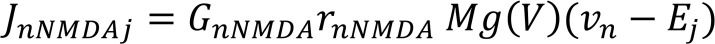
Equation 8

In Equations 7 and 8, *j* represents Na^+^, K^+^, or Ca^2+^ ion (only for NMDA). *G_nAMPA_/G_nNMDA_*, *r_nAMPA_*, and *r_nNMDA_* represent the maximal conductance of AMPA/NMDA receptor channels, the open probability of AMPA, and NMDA receptors, respectively. The equations for *r_nAMPA_* and *r_nNMDA_* are given as in Equation 9.


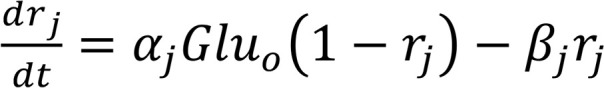
Equation 9

In Equation 9, term *j* represents AMPA or NMDA receptor, and *α_j_* and *β_j_* are the activation and inactivation rates.

*Mg* (*v_n_*) denotes a voltage-dependent magnesium ([*mg*]) block modeled with Equation 10.


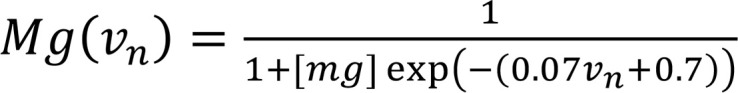
Equation 10

The flux through NKCC1 is given by Equation 11 (44).

,
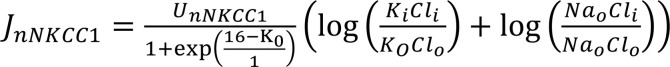
Equation 11

where *U_nKCC1_* is the maximum flux due to NKCC1 transporter.

Neuronal calcium ([Ca^2+^]_ni_) is regulated by fluxes through NCX1, NMDA receptors (*J_NMDACa_*), and VGCCs (*J_VGCC_*) according to Equation 12.


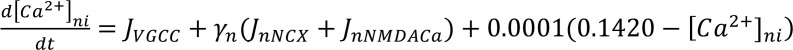
,Equation 12

where

.
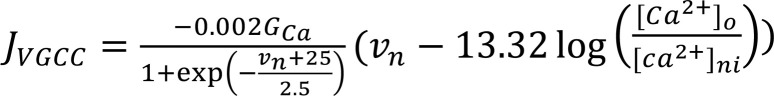
Equation 13

Here, *G_Ca_* is the maximum conductance of neuronal VGCCs. The other 2 fluxes are as described in the supplemental material.

### Numerical methods.

The rate equations were solved in Python 3.10.9 using the Euler method with a time step of 0.1 second. The system of equations was allowed to reach steady state before initiating ischemic condition.

### Data analysis and statistics.

All data analysis was conducted blinded. Time-lapse series were imported into ImageJ 2.0 (NIH) and stabilized using the Image Stabilizer plugin for ImageJ (K. Li, Carnegie Mellon University, Pittsburgh, PA; https://imagej.net/plugins/image-stabilizer). Regions of interest (ROIs) were defined manually. For calcium analysis, fluorescence over time was defined for each neuronal or astroglial ROI, converted to ΔF/F, and imported into Matlab (MathWorks). We used custom-written algorithms to smooth the signal with median and Gaussian filters. Subsequently, peak amplitude, time to peak, peak to baseline, and full duration at half maximum (FDHM) were determined for each signal using a custom-written algorithm in Matlab. Calcium changes during spreading depolarizations were defined as signals when fluorescence exceeded ≥2 SD relative to baseline fluorescence. ΔF/F in each ROI was plotted together with the respective video file for visual inspection and verification. The velocity of calcium signal propagation was calculated by dividing the distance between 2 ROIs by the time lag between the onset of calcium signals at these points. For sodium analysis, fluorescence intensity changes in neurons and astrocytes were determined and analyzed as described above (astrocytes were distinguished from neurons by their SR101 colabeling). For glutamate analysis, ROIs corresponding to individual SF-iGluSnFR–expressing astrocytes were manually identified using ImageJ. Intensity changes over time for each ROI were determined and analyzed as described above.

Immunohistochemical data were analyzed by binarizing confocal NCX1 images using a threshold based on the average background intensity plus the triplicate of the standard deviation. A cell was defined as NCX1-positive if 6 or more punctuate NCX1 signals colocalized with either the GFAP or the NeuN counterstain, respectively.

Data were excluded from the analysis if an animal died during an experiment. No outliers were excluded from the datasets. All studies were performed by investigators blinded to genotype. We used the 2-tailed Mann-Whitney test for comparisons between 2 groups and Kruskal-Wallis test followed by Dunn’s multiple-comparison test for comparisons between 3 groups. Tukey’s box-and-whisker plots indicate the median (line), interquartile range (IQR, box), and 1.5 IQR (whiskers) in all figures. Data were analyzed using Prism 10 (GraphPad) and are represented as mean ± SEM. A *P* value of less than 0.05 was accepted as statistically significant.

### Study approval.

All experimental procedures were reviewed and approved by local authorities (Landesamt für Verbraucherschutz und Ernährung North Rhine-Westphalia, Recklinghausen, Germany).

### Data availability.

Values for each data point presented in the graphs can be found in the supplemental Supporting Data Values file.

## Author contributions

Conceptualization: GCP; Methodology: SHT, PKT, CR, NJG, CRR, GU, and GCP; Investigation: SHT, PKT, and NJG; Visualization: SHT and GCP; Funding acquisition: GCP and SRR; Supervision: GCP; Writing – original draft: GCP; Writing – review and editing: SHT, GU, and CRR.

## Conflict of interest

The authors have declared that no conflict of interest exists.

## Funding support

German Science Foundation (Deutsche Forschungsgemeinschaft, DFG) FOR 2795 “Synapses Under Stress” grants PE1193/6-2 and RO2327/13-2 (to GCP, CRR, and GU).

- Mercator fellowship (to GU).
- ERA-NET (MICRO-BLEEDs and TACKLE-CSVD).
- Fondation Leducq (Transatlantic Network of Excellence 23CVD03).

## Supplementary Material

Supplemental data

Supporting data values

## Version 1 06/02/2026

In-Press Preview

## Version 2 07/22/2026

Electronic publication
