# Supplementary material for: Neuronal and astrocytic sodium-calcium exchanger differentially regulates calcium and sodium overload during ischemic stroke
Source: JCI Insight. 2026 Jun 2;11(14):e200411. doi: 10.1172/jci.insight.200411 (PMC13461163; doi:10.1172/jci.insight.200411)
Supplement: Supplementary file 1 [file jciinsight-11-200411-s152.pdf]

Supporting Information: Neuronal and astrocytic sodium-calcium exchanger differentially regulates calcium and sodium overload during ischemic stroke

Ion dynamics in ECS and neurons/astrocyte

K^+ concentration in the ECS (K^+) due to astrocytes depends upon the fluxes of Na^+/K^+-ATPase pump, K^+ channels, co-transporter as well as K^+ exchange with the bath solution. The rate equation for extracellular K^+ concentration is

$$\frac{d[K^+]_{SCa}}{dt} = \beta_a\Big(J_{aK} - 2J_{aNaK} - J_{aNKCC} - J_{aKCC} + \big(-J_{aEAAT2rev}\big) \times \gamma_a\Big) + J_{Kdiff}, \tag{1}$$

where β_a is the volume ratio between astrocytic to ECS region. The flux through the K^+ channel $(\mu M s^{-1})$ is

$$J_{aK} = G_{aK}\left(v - E_K\right). \tag{2}$$

G_K is whole-cell conductance of K^+, v is the membrane potential, and E_K is the reversal potential of K^+ channel. Nernst potential for the K^+ channel (mV) is

$$E_x = \frac{v_T}{z}\ln\left(\frac{[x]_o}{[x]_{zi}}\right), \tag{3}$$

where, x is Na^+,K^+ or Cl^- and z is neuron or astrocytes and $v_T = \frac{RT}{F}$.

Na^+/K^+-ATPase exchanges 3 Na^+ to exit the astrocyte for the influx of 2 K^+. The flux through the Na^+/K^+ pump $(\mu M s^{-1})$ is

$$J_{NaKa} = \phi(t) J_{NaKa_{max}} H_{1.5}\big([Na^+]_{ai}, K_{[Na^+]_{ai}}\big) H\big([K^+]_o, [K^+]_{[K^+]_o}\big). \tag{4}$$

$\phi(t)$ is 0.5 for $0 < t < 20$s and 1 otherwise. $J_{NaK_{max}}$ is a maximum flux through Na^+/K^+ pumps.

One Na^+, one K^+, and 2 Cl^- ions move in inward direction through NKCC1. The flux through NKCC $(\mu M s^{-1})$ is

$$J_{aNKCC1} = G_{aNKCC1} v_T \ln\left(\frac{[Na^+]_o[K^+]_o[Cl^-]_o^2}{[Na^+]_{ai}[K^+]_{ai}[Cl^-]_{ai}^2}\right). \tag{5}$$

G_{aNKCC1} is the whole-cell conductance of NKCC.

Cl^- and K^+ flux through the KCC channel $(\mu M s^{-1})$:

$$J_{aKCC} = G_{aKCC} v_T \ln\left(\frac{[K^+]_o[Cl^-]_o}{[K^+]_{ai}[Cl^-]_{ai}}\right). \tag{6}$$

G_{aKCC} is the whole-cell conductance of KCC

K^+ flux due to glutamate transporters is

$$J_{aEAAT2rev} = J_{aEAAT2revmax} H\big([K^+]_o, [K^+]_{aEAAT2mK}\big) H_3\big([Na^+]_{ai}, K_{aEAAT2mN}\big) H\big(glu_{ai}, K_{aEAAT2mg}\big) H\big([H^+]_{ai}, K_{aEAAT2mH}\big). \tag{7}$$

$J_{aEAAT2revmax}$ is the maximum current through the astrocyte reverse glutamate transporters. $K_{aEAAT2mK}$, $K_{aEAAT2mN}$, and $K_{aEAAT2mg}$ represent the half-saturation constant for K^+, Na^+, and glutamate, respectively.

γ_a converts pA/um^2 to uM/sec and is given as

$$\gamma_a = 10^3 \frac{A_a}{\left(F \times vol_a\right)}. \tag{8}$$

A_a is the area of the astrocyte, F is Faraday's constant, and vol_a is the astrocyte volume.

K^+ diffusion between ECs and bath solution $\left(\mu M s^{-1}\right)$ is

$$J_{Kdiff} = diff([K^+]_{bath} - [K^+]_o), \tag{9}$$

where $diff$ is the diffusion constant.

The rate equations for the Na^+ concentration in the ECS is modeled as

$$[Na^+]_o = 149 + \beta_a(12 - [Na^+]_{ai}) \tag{10}$$

Cl^- concentration in the ECS is (μM) is given by conservation of charge, that is

$$[Cl^-]_o = [Na^+]_o + [K^+]_o. \tag{11}$$

K^+ concentration in the astrocyte (K_a) is defined as.

$$[K^+]_{ai} = 146 + (12 - [Na^+]_{ai}) \tag{12}$$

$[Na^+]_{ai}$ depends on the flux through Na^+ channels, Na^+/K^+ ATPase, NKCC, NCX, and glutamate transporters. Some of them are already explained in the above sections.

$$\begin{aligned} \frac{d[Na^+]_{ai}}{dt} = -J_{aNa} - 3J_{aNaK} + J_{aNKCC} - \\ \left(3J_{aNCX} + 3J_{aEAAT2rev}\right) \times \gamma_a. \end{aligned} \tag{13}$$

Na^+ flux through Na^+ channels $(\mu M s^{-1})$:

$$J_{aNa} = G_{Na}\left(v - E_{aNa}\right). \tag{14}$$

G_{aNa} is the whole-cell conductance of astrocytic Na^+ channels and E_{aNa} is the reversal potential for the Na^+.

$$E_{Na} = \frac{v_T}{z_{Na}} \ln\left(\frac{[Na^+]_o}{[Na^+]_{ai}}\right). \tag{15}$$

The intracellular Na^+ also depends upon flux due to astrocytes NCX, which is given by

$$\begin{aligned} J_{aNCX} = J_{aNCXmax} H_3\left([Na^+]_o, K_{NCXmN}\right) H\left([Ca^{2+}]_o, K_{NCXmC}\right) \times \\ \frac{\frac{[Na^+]_{ai}^3}{[Na^+]_o^3} exp\left(\eta_{NCX} \times \frac{v_a}{v_T}\right) - \frac{[Ca^{2+}_{ai}]}{[Ca^{2+}]_o} exp\left((\eta_{NCX} - 1) \times \frac{v_a}{v_T}\right)}{1 + k_{sat} exp\left((\eta_{NCX} - 1) \times \frac{v_a}{v_T}\right)}, \end{aligned} \tag{16}$$

where $J_{aNCXmax}$ is the maximum current through astrocytes NCX. K_{NCXmN}, and K_{NCXmC} are the binding affinities of Na^+ and Ca^{2+}, respectively, to NCX.

Na^+ flux through glutamate transporters is obtained by multiplying the K^+ flux due to transporters by a factor of 3 because the stoichiometry of EAAT is 3 Na^+: K^+:glu:H^+

Cl$^-$ concentration in the astrocyte is given by electroneutrality (μM).

$$\frac{d[Cl^-]_{ai}}{dt} = \frac{d[Na^+]_{ai}}{dt} + \frac{d[K^+]_{ai}}{dt} + 2\frac{d[Ca^{2+}]_{ai}}{dt}, \tag{17}$$

Cytosolic Ca^{2+} ($[Ca^+]_{ai}$) is regulated by IP_3 receptors-mediated release from the endoplasmic reticulum (ER), release from the ER through leak channels, uptake by the ER through SERCA, influx from ECS through NMDA receptors, and flux through NCX.

$$\frac{d[Ca^{2+}]_{ai}}{dt} = B_{cyt}\Big(J_{IP3} - J_{pump} + J_{ERleak}\Big) + \big(J_{NCX}\big) \times \gamma_a. \tag{18}$$

The Ca^{2+} buffering is described by steady-state approximation, i.e.

$$B_{cyt} = \left(1 + BK_{end} + \frac{K_{ex}B_{ex}}{(K_{ex} + [Ca^{2+}]_{ai})^2}\right)^{-1}, \tag{19}$$

where BK_{end} is the ratio of endogenous buffer concentration to dissociation constant, K_{ex} is dissociation constant of exogenous buffer, and B_{ex} the concentration of an exogenous buffer.

The flux of Ca^{2+} through the IP_3R channels $(\mu M s^{-1})$ is

$$J_{IP3} = J_{max}\Big(H\big(IP_3, [K^+]_i\big)H\big([Ca^{2+}]_{ai}, [K^+]_{act}\big)h\Big)^3\left(1 - \frac{[Ca^{2+}]_{ai}}{s}\right). \tag{20}$$

J_{max} is the maximum rate of Ca^{2+} through IP_3Rs, K_i dissociation constant for IP_3 binding to IP_3R, K_{act} dissociation constant for Ca^{2+} binding to an activation site on the IP_3R.

Ca^{2+} flux through SERCA pumps $(\mu M s^{-1})$ is

$$J_{pump} = V_{max}H_2\big([Ca^{2+}]_{ai}, K_{pump}\big). \tag{21}$$

V_{max} is the maximum rate of Ca^{2+} uptake by SERCA pumps and k_{pump} represents the Ca^{2+} dissociation constant of SERCA.

Ca^{2+} flux through the leak channels $(\mu M s^{-1})$ is

$$J_{ERleak} = P_L\left(1 - \frac{[Ca^{2+}]_{ai}}{s}\right). \tag{22}$$

P_L is the maximum flux through leak channels.

Ca^{2+} flux through NCX is 3 times smaller than the Na^+ given by $(eq.16)$ due to 3:1 stoichiometry. That is, NCX exchanges 3 Na^+ for 1 Ca^{2+}.

The astrocytic inositol 1,4,5-trisphosphate IP_3 concentration (μM) is given by the following rate equation.

$$\frac{dIP_3}{dt} = r_hG - k_{deg}IP_3. \tag{23}$$

r_h is the maximum rate of IP_3 production in astrocytes due to metabotropic glutamate receptors and K_{deg} is the rate constant for IP_3 degradation. The ratio of active to total G-proteins (G) is

$$G = \frac{\rho + \delta_G}{K_G + \rho + \delta_G}. \tag{24}$$

The ratio ρ of bound to unbound metabotropic receptors on the astrocytic process is

$$\rho = \rho_{min} + \frac{\rho_{max} - \rho_{min}}{glu_{max}} glu_o. \tag{25}$$

glu_o is the extracellular glutamate concentration described in method sections. We apply 1 mM for 100 ms, which decays exponentially as discussed in the main text.

The astrocytic epoxyeicosatrienoic acid (EET) concentration (μM) is modeled by the following equation.

$$\frac{deet}{dt} = V_{eet} max\left([Ca^{2+}]_{ai} - c_{k_{min}}, 0\right) - k_{eet} eet \tag{26}$$

V_{eet} and k_{eet} are the EET production and degradation rates, respectively.

Ca^{2+} concentration in the ER (μM) is

$$\frac{ds}{dt} = \frac{-B_{cyt}}{VR_{ER_{cyt}}} \left(J_{IP3} - J_{pump} + J_{ERleak}\right). \tag{27}$$

$VR_{ER_{cyt}}$ is the volume ratio between ER and cytosol.

The inactivation variable h of IP_3R channels is

$$\frac{dh}{dt} = k_{on}\left[K_{inh} - \left([Ca^{2+}]_{ai} + K_{inh}\right) h\right], \tag{28}$$

where k_{on} is the rate of Ca^{2+} binding to the inhibitory site of IP_3R and K_{inch} is dissociation constant of inhibitory site.

Membrane potential of the astrocyte (mV) is

$$\begin{aligned}\frac{dv_a}{dt} = \gamma_v\left(-J_K - J_{Cl} - J_{Na} - J_{NaK}\right) - \\ \left(-2J_{EAAT2rev} + J_{NCX}\right) \times \gamma_a,\end{aligned} \tag{29}$$

where γ_v converts flux from concentration unit to current unit. The fluxes involve in the astrocytic membrane potential except J_{Cl} are already explained in the above section.

Cl^- flux through leak channels $(\mu M s^{-1})$ is

$$J_{Cl} = G_{Cl}\left(v - E_{Cl}\right). \tag{30}$$

G_{Cl} is the maximum conductance of Cl^- channels. E_{Cl} is the reversal potential of Cl^- and is given by

$$E_{Cl} = \frac{v_T}{z_{Cl}} \ln\left(\frac{[Cl^-]_o}{[Cl^-]_{ai}}\right), \tag{31}$$

z_{cl} is the valence of Cl^-.

Number of K^+ concentration in the ECS depends upon the fluxes of voltage gated K^+ channels, Na^+/K^+ATPasepump, glia, glialpump,co-transporter, NMDA and AMPA receptors. The rate equations for the extracelluar potassium is

$$\frac{d[K^+]_o}{dt} = \frac{1}{\tau}\left(\gamma_n\beta_n(J_{nK} + J_{nNMDAK} + J_{nAMPAK}) - 2\beta J_{\text{npump}} - J_{\text{diff}} + \beta_n J_{\text{nkcc2}} + \beta_n J_{\text{nnkcc1}} + FluxaK10^{-3}\right) \tag{32}$$

where τ convert to ms, $\gamma_n = S/(F\nu_i)$ is a conversion factor from current units (μA/cm^2) to mM/s. $\beta_n = \frac{\nu_{ni}}{\nu_o}$ is the intra to extracellular volume ratio.

The potassium current passing through the voltage gated potassium channel is

$$J_{\text{nK}} = G_{\text{nK}} n^4 (V - E_{\text{K}}) + G_{\text{KL}} (V - E_{\text{K}}) \tag{33}$$

where, G_K and G_{KL} represent K^+ voltage-gate maximal and leak conductance respectively The equaitons for the neuronal Na^+K^+-ATPase pump is

$$J_{\text{npump}} = \rho \times \frac{1}{1 + \exp\left(\frac{25 - [\text{Na}^+]_i}{3}\right)} \times \frac{1}{1 + \exp\left(3.5 - [\text{K}^+]_o\right)} \tag{34}$$

where ρ represent pump strength and its equation is

$$\rho = \frac{\rho_{\text{max}}}{1 + \exp\left(\frac{20 - [\text{O}_2]_o}{3}\right)} \tag{35}$$

ρ_{max} represent maximum pump strength

The flux due to K^+Cl^- cotransporter 2 is

$$J_{\text{nkcc2}} = U_{\text{nkcc2}} \ln\left(\frac{[\text{K}^+]_o}{[\text{K}^+]_i}\right) \tag{36}$$

where U_{kcc2} is the cotransporter strength

The flux due to $Na^+K^+Cl^-$ transporter 1 is

$$J_{\text{nnkcc1}} = U_{\text{nnkcc1}} f\left([\text{K}^+]_o\right) \ln\left(\frac{[\text{Na}^+]_i[\text{Cl}^-]_i}{[\text{Na}^+]_o[\text{Cl}^-]_o}\right) \tag{37}$$

where U_{nkcc1} is the cotransporter strength

$$f\left([\text{K}^+]_o\right) = \frac{1}{1 + \exp\left(16 - [\text{K}^+]_o\right)} \tag{38}$$

The equations for activation and inactivation variable is

$$\frac{dq}{dt} = \alpha_q (1 - q) - \beta_q q, \quad q = m, h, n', \tag{39}$$

The equations for the closing and opening rate of the activatons and inactivations variables are represented by α and β of respective varibles. Their equations are

$$\alpha_m = \frac{0.32(V + 54)}{1 - \exp\left(-\frac{V+54}{4}\right)} \tag{40}$$

$$\beta_m = \frac{0.28(V + 27)}{\exp\left(\frac{V+27}{5}\right) - 1} \tag{41}$$

$$\alpha_h = 0.128 \exp\left(-\frac{V + 50}{18}\right) \tag{42}$$

$$\beta_h = \frac{4}{1 + \exp\left(-\frac{V+27}{5}\right)} \tag{43}$$

$$\alpha_n' = \frac{0.032(V+52)}{1 - \exp\left(-\frac{V+52}{5}\right)} \tag{44}$$

$$\beta_n' = 0.5 \exp\left(-\frac{V+57}{40}\right) \tag{45}$$

The equation of the flux due to potassium diffusion current is

$$J_{\text{diff}} = \epsilon_k \left([\text{K}^+]_o - [\text{K}^+]_{\text{bath}}\right) \tag{46}$$

ϵ_k is the diffusion of K^+ to the blood whose equations is

$$\epsilon_k = \frac{1}{1 + \exp\left(\frac{-20+\beta_n}{2}\right)} \times \frac{\epsilon_{k,\text{max}}}{1 + \exp\left(\frac{-[\text{O}_2]_{\text{bath}} - 2.5}{0.2}\right)} \tag{47}$$

$\epsilon k, max$ is the maximum diffusion of $K^+$to blood. $[K^+]_{\text{bath}}$ and $[O_2]_{\text{bath}}$ are the bath concentrations of K^+ and O_2

The fluxes due to neuronal AMPA and NMDA receptors are modeled as

$$J_{\text{nAMPAj}} = G_{nAMPA} r_{nAMPA} (v_n - E_j) \tag{48}$$

$$J_{\text{nAMPAj}} = G_{nNMDA} r_{nNMDA} Mg(V)(v_n - E_j), \tag{49}$$

Where j represents Na^+,K^+, or Ca^{2+} ion (only for NMDA). $G_{\text{nAMPA}}/G_{\text{nNMDA}}$, r_{nAMPA}, and r_{nNMDA} represent the maximal conducatnace of AMPA/NMDA receptor channels, the open probabiity of AMPA, and NMDA receptors, respectively. The equations for r_{nAMPA}, and r_{nNMDA} are given as

$$\frac{dr_j}{dt} = \alpha_j [Glu]_o (1 - r_j) - \beta_j r_j \tag{50}$$

where j represents AMPA or NMDA receptor, α_j and β_j are the activation and inactivation rates. $Mg(v_n)$ denotes a voltge-dependent magnesium ([mg]) block modeled with the follwoing equaitons

$$Mg(v_n) = \frac{1}{1 + [mg] exp(-(0.07 v_n + 0.7))} \tag{51}$$

FluxaK is the flux due to astrocytes potassium concentrations given by

$$Fluxak = \beta_a (J_{aK} - 2J_{aNaK} - J_{aNKKC1} - J_{KCC1} - J_{aEAAT2rev} \gamma_a) \tag{52}$$

The K^+ concentrations in the neurons depends on voltage gated K^+ channels, NMDA and AMPA receptors, Na^+K^+-ATPase pump, KCC2, KCC1. The equations for rate of change of intracellular K^+ ions is

$$\frac{d[K^+]_{ni}}{dt} = \frac{1}{\tau} \left(-\gamma (J_K + J_{nNMDAK} + J_{nAMPAK}) + 2I_{\text{npump}} - J_{\text{nkcc2}} - J_{\text{nnkcc1}}\right) \tag{53}$$

The detail explanations of the calculations of NMDA and AMPA receptors current due to potassium is already described in the method sections of the manuscript

The Na^+ concentrations in the ECS is due to the fluxes through voltage gated Na^+ channels, Na^+K^+-ATPase pump, NMDA and AMPA receptors, NCX and NKCC1, The equations for the rate of change of extracellular Na^+ ions is

$$\frac{d[Na^+]_o}{dt} = \frac{1}{\tau}\left(\gamma_n\beta_n(J_{\text{nNa}} - J_{nNMDANa} - J_{nAMPANa} - J_{nNCX}) + 3\beta J_{\text{npump}} + \beta J_{\text{nnkcc1}} + FluxaNa10^{-3}\right) \tag{54}$$

Sodium current passing through the voltaged gated sodium channel is

$$J_{\text{nNa}} = G_{\text{Na}}m^3h(V - E_{\text{Na}}) + G_{\text{Nal}}(V - E_{\text{Na}}) \tag{55}$$

where, G_{Na} represent Na^+ voltage-gate maximal conductance. The fluxes of NMDA and AMPA receptors are calculated as explained in method sections. We used similar expression of NCX flux as in astrocytes to explain the neruonal NCX flux with variations of maxium NCX current.

FluxaNa is the flux due to astrocytes Na^+ concentrations given by

$$FluxaNa = \beta_a(J_{aNa} + 3J_{aNaK} - J_{aNKKC1} + (3J_{aNCX} + 3J_{aEAAT2rev})\gamma_a) \tag{56}$$

$[Na^+]_{ni}$ depends upon the fluxes of J_{nNa}, $J_{nNMDANa}$, $J_{nAMPANa}$, J_{nNCX}, J_{npump} and J_{nnkccl} The equations for the rate of change of intracellular Na^+ ions is

$$\frac{d[Na^+]_{ni}}{dt} = \frac{1}{\tau}\left(-\gamma_n(J_{\text{nNa}} - J_{nNMDANa} - J_{nAMPANa} - J_{nNCX}) - 3J_{\text{npump}} - J_{\text{nnkcc1}}\right) \tag{57}$$

Neuronal calcium ($[Ca^{2+}]_{ni}$)is regulated by fluxes through NCX1, NMDA receptors (J_{NMDACa}) and VGCC(J_{VGCC})

$$\frac{d[Ca^{2+}]_{ni}}{dt} = J_{VGCC} + \gamma_n(J_{\text{nNCX}} + J_{nNMDACa}) + 0.0001\left(0.1420 - [Ca^{2+}]_{ni}\right) \tag{58}$$

Where,

$$J_{\text{VGCC}} = \frac{-0.002G_{Ca}}{1 + exp\left(-\frac{v_n+25}{2.5}\right)}\left(v_n - 13.2log\left(\frac{[Ca^{2+}]_o}{[Ca^{2+}]_{ni}}\right)\right) \tag{59}$$

Here, G_{Ca} is the maximum conducatiance of neuronal VGCCs.

Extracellular Cl^- depends on the fluxes due to leaked chlorine channels Cll, NKCC2, and KCCl1. The equations for the rate of change of extracellular Cl^- ions is

$$\frac{d[Cl_o]}{dt} = \frac{1}{\tau}\left(-\gamma_n\beta_n J_{\text{nCll}} + \beta_n J_{\text{nnkcc2}} + 2\beta J_{\text{nkcc1}}\right) \tag{60}$$

The chloride leak current is

$$J_{\text{nCl}} = G_{\text{nCll}}(V - E_{\text{Cl}}) \tag{61}$$

G_{nClL} represent leak conductance

The equations for the rate of change of number of intracellular Cl^- ions is

$$\frac{d[Cl_i]}{dt} = \frac{1}{\tau}\left(\gamma J_{\text{Cll}} - J_{\text{kcc2}} - 2J_{\text{nkcc1}}\right) \tag{62}$$

$$\frac{d[\text{O}_2]_o}{dt} = -\alpha\left(J_{\text{npump}} + J_{\text{aNaK}}10^{-3}\right) + \epsilon_o\left([\text{O}_2]_{\text{bath}} - [\text{O}_2]_o\right) \tag{63}$$

where α converts charge utilization in the pump current (mM/s) to the rate of oxygen concentration change ($\text{mgL}^{-1}\text{s}^{-1}$), $[O_2]_{\text{bath}}$ is the bath oxygen concentration in the perfusion solution, and ϵ_o is a diffusion constant obtained from Ficks's law.

$$\epsilon_o = \frac{D}{\Delta x^2} \tag{64}$$

Where D is diffussion coeffiecent and Δx is the average distance from the electrode tip

The equation for the initial neuronal or astroytes volume is

$$\hat{\nu}_{xi} = \nu_{xi0} \times \left(1.1029 - 0.1029 \times \exp\left(\frac{\pi_o - \pi_{xi}}{20}\right)\right) \tag{65}$$

where x is n or a.

The equaiton for the extracellular osmotic pressure is

$$\pi_o = [\text{Na}^+]_o + [\text{K}^+]_o + [\text{Cl}^-]_o + [\text{A}^-]_o \tag{66}$$

The equation for the intracellular osmotic pressure is

$$\pi_{xi} = [\text{Na}^+]_{xi} + [\text{K}^+]_{xi} + [\text{Cl}^-]_{xi} + [\text{A}^-]_{xi} \tag{67}$$

where $[A]_i$ and $[A]_o$ are the intra- and extra-cellular concentration of the impermeable anions. The equations for the intracellular volume is

$$\frac{d\nu_{xi}}{dt} = \frac{\hat{\nu}_{xi} - \nu_{xi}}{250} \tag{68}$$

The equations for the extracellular volume is

$$\nu_o = \left(1 + \frac{1}{\beta_0}\right)\nu_{xi0} - \nu_{xi} \tag{69}$$

Membrane potential of the neurons (mV) is

$$C\frac{dV}{dt} = -J_{\text{Na}} - J_{\text{K}} - J_{\text{Cl}} - \frac{J_{\text{pump}}}{\gamma_n} \tag{70}$$

Table S1: Parameters for ion dynamics in the astrocyte and ECS and membrane potential of the astrocyte.

Parameter	Description	value
VR_{sa}	Volume ratio between the ECS and astrocyte	3
glu_{max}	Maximum glutamate concentration	1000 μM
G_{aK}	Peak conductance of K^+ channels	6907.77 $\mu M mV^{-1}s^{-1}$
G_{aNa}	Peak conductance of Na^+ channels	226.94 $\mu M mV^{-1}s^{-1}$
G_{aKCC}	Peak conductance of the KCC	1.728 $\mu M mV^{-1}s^{-1}$
G_{aNKCC}	Peak conductance of the NKCC	9.568 $\mu M mV^{-1}s^{-1}$
G_{aCl}	Peak conductance of Cl^{-1} leak	151.93 $\mu m V^{-1}s^{-1}$
$J_{aNaK_{max}}$	Maximum flux through Na^+/K^+ ATPase in the cortex	2.84×10^4 $\mu M s^{-1}$
$J_{aNaK_{max}}$	Maximum flux through Na^+/K^+ ATPase in the hippocampus	2.934×10^4 $\mu M s^{-1}$
K_{aNa}	Association constant for Na^+ to Na^+/K^+ATPase	10×10^3 μM
K_{K_o}	Association constant for K^+ to Na^+/K^+ ATPase	1.5×10^3 μM
ρ_{min}	Minimum ratio of bound to unbound IP_3 receptors	0.1
ρ_{max}	Maximum ratio of bound to unbound IP_3 receptors	0.7
δ_G	Ratio of the activities of the unbound and bound receptors	1.235×10^{-2}
K_G	G-protein disassociation constant	8.82
r_h	Maximum rate of IP_3 production due to mGluRs	4.8 $\mu M s^{-1}$
K_{deg}	Rate constant in for IP_3 production	1.25 s^{-1}
r_{buff}	Rate of Ca^{2+} buffering at the endfeet compared to the astrocyte body	0.05
$VR_{ER_{cyt}}$	Volume ratio between the ER and astrocytic cytosol	0.185
BK_{end}	Ratio of endogenous buffer concentration to disassociation constant	40
K_{ex}	Disassociation constant of exogenous buffer	0.26 μM
B_{ex}	Concentration of exogenous buffer	11.35 μM
J_{max}	Maximum rate of Ca^{2+} through the IP_3Rs	2880 $\mu M s^{-1}$
K_i	Disassociation constant of IP_3 binding to IP_3R	0.03 μM
K_{act}	Disassociation constant of Ca^{2+} binding to the activation site of IP_3R	0.17 μM
k_{on}	Rate of Ca^{2+} binding to the inhibitory site of IP_3R	2 $\mu M^{-1}s^{-1}$
K_{inch}	Disassociation constant of IP_3R	0.1 μM
V_{max}	Maximum rate of SERCA	20 $\mu M s^{-1}$
k_{pump}	Disassociation constant of SERCA	0.24 μM
P_L	ER leak channel steady-state balance constant	0.0804 $\mu M s^{-1}$
V_{eet}	EET production rate	72 s^{-1}
c_{min}	Minimum Ca^{2+} concentration required for EET production	0.1 μM
k_{eet}	EET degradation rate	7.2 S^{-1}
v_4	Measure of the spread of W_∞	8 mV
eet_{shift}	Describe the EET dependent voltage shift	2 $mV \mu M^{-1}$
Na_{bath}	Bath concentrations of Na^+	152 mM
K_{bath}	Bath concentration of K^+	2.6 mM
$diff$	Diffusion constant of Na^+ and K^+	0.5 s^{-1}

Table S2: Parameters for glutamate transporters and NCX.

Parameter	Description	value
$gluo, eq$	extracellular glutamate concentrations at equilibrium	$0.25uM$
glu_{ni}	neuronal release of glutamate	$1mM$
$[Ca^{+}]_{athr}$	threshold value of the Ca^{2+} for the vesicular release of the glutamate	$0.4uM$
r_{aglu}	Probability of vesicular release rate	0.2
glu_{v}	Releaseable glutamte from vesicle	$r_{aglu}glu_{ni}$
$J_{aEAAT2revmax}$	Maximum glutamate transporters flux	1.03 $pA\mu m^{-2}$
$K_{aEAAT2mN}$	Rate of sodium binding to glutamate transporter	15000 μM
$K_{aEAAT2mK}$	Rate of potassium binding to glutamate transporter	5000 μM
$K_{aEAAT2mg}$	Rate of glutamate binding to glutamate transporter	34 μM
$K_{aEAAt2mH}$	Rate of Proton binding to glutamte transporter	$300nM$
$J_{aNCX_{max}}$	Maximum NCX current	0.001 $pA\mu m^{-2}$
K_{aNCXmN}	Half-saturation constant for sodium	875000 μM
K_{aNCXmC}	Half-saturation constant for calcium	1380 μM
η_{aNCX}	The position of energy barrier	0.35
$ksat$	Saturation factor	0.1

Table S3: The initial values of various variables.

Parameter	Description	value
$[Ca^{2+}]_{o}$	Extracellular calcium concentration	2000 μM
$[Ca^{2+}]_{ai}$	Intracellular calcium concentration	0.12 μM
$[Na^{+}]_{o}$	Extracellular Na^{+} concentration	149 mM
$[Na^{+}]_{ai}$	Intracellular Na^{+} concentration	14 mM
$[K^{+}]_{o}$	Extracellular K^{+} concentration	2.5 mM
$[K^{+}]_{ai}$	Intracellular K^{+} concentration	146 mM
$[Cl^{+}]_{ai}$	Intracellular Cl^{-} concentration	7.6 mM
v_{a}	Intracellular membrane potential	-89 mV
IP_{3}	Initial IP_{3} concentration	0.048 μM
EET_{a}	Initial EET concentration	0.70 μM
h_{a}	Initial value of the inactivation variable	0.55
s_{a}	Initial calcium concentration in astrocytic ER	466 μM

Table S4: Parameters for ion dynamics in the neurons.

Parameter	Description	value
v_{n0}	Resting membrane potential of the neuron	$-70mV$
$[K^+]_{ni0}$	Initial neuronal K^+ concentrations	$140mM$
$[Na^+]_{ni0}$	Initial neuronal Na^+ concentrations	$18.0mM$
$[Cl^-]_{ni0}$	Initial neuronal chloride concentration	$6mM$
K_{bath}	Bath concentrations of the potassium	$7.5mM$
O_{bath}	Bath concentrations of the oxygen	$32mgL^{-1}$
K_{bathSD}	Bath concentrations of the potassium at SD	$20mM$
O_{bathSD}	Bath concentrations of the oxygen at SD	$20mgL^{-1}$
G_{nK}	Voltage-gated potassium conductance	25.0 $mScm^{-2}$
G_{nNa}	Voltage-gated sodium conductance	30.0 $mScm^{-2}$
G_{nCll}	Leak chloride conductance	0.1 $mScm^{-2}$
G_{nKl}	Leak potassium conductance	0.05 $mScm^{-2}$
G_{nNal}	Leak sodium conductance	0.0247 $mScm^{-2}$
C	Membrane capacitance	1.0 μFcm^{-2}
$G_{glia,max}$	Maximal glial upadte strength of potassium	5 mMs^{-1}
$\epsilon_{k,max}$	Maximal potassium diffusion rate	.25 s^{-1}
ϵ_o	Oxygen diffusion coefficient	0.17 s^{-1}
U_{nKCC2}	Maximal KCC2 cotransporter strength	0.3 mMs^{-1}
U_{nNKCC1}	Maximal NKCC1 cotransporter strength	0.1 mMs^{-1}
ρ_{max}	Maximal pump rate	0.8 mMs^{-1}
v_{io}	Initial intracellular volume	1.4368×10^{-15} m^3
β_0	Ratio of initial intra-/extracellular volume	7.0
$[A]_o$	Extracellular concentrations of the impermeable ions	18 mM
$[A]_i$	Intracellular concentrations of the impermeable ions	132 mM
τ	Time constant	0.001 s
Glu_o	Glutamate concentration used for NMDA and AMPA receptors activation in neurons	1 mM
$[Ca^{2+}]_{n0}$	Initial calcium concentratoins in neurons	$0.14uM$
G_{AMPA}	Maximal conductance of AMPARs	$0.0145mScm^{-2}$
G_{NMDA}	Maximal conductance of NMDARs	$0.026mScm^{-2}$
$[Mg]$	Magnesium concentration	1 mM
α_{AMPA}	Binding rate of AMPARs	$1.1mMms^{-1}$
α_{NMDA}	Binding rate of NMDARs	$0.072mMms^{-1}$
β_{AMPA}	Unbinding rate of AMPARs	$0.19ms^{-1}$
β_{NMDA}	Unbinding rate of NMDARs	$0.0066ms^{-1}$

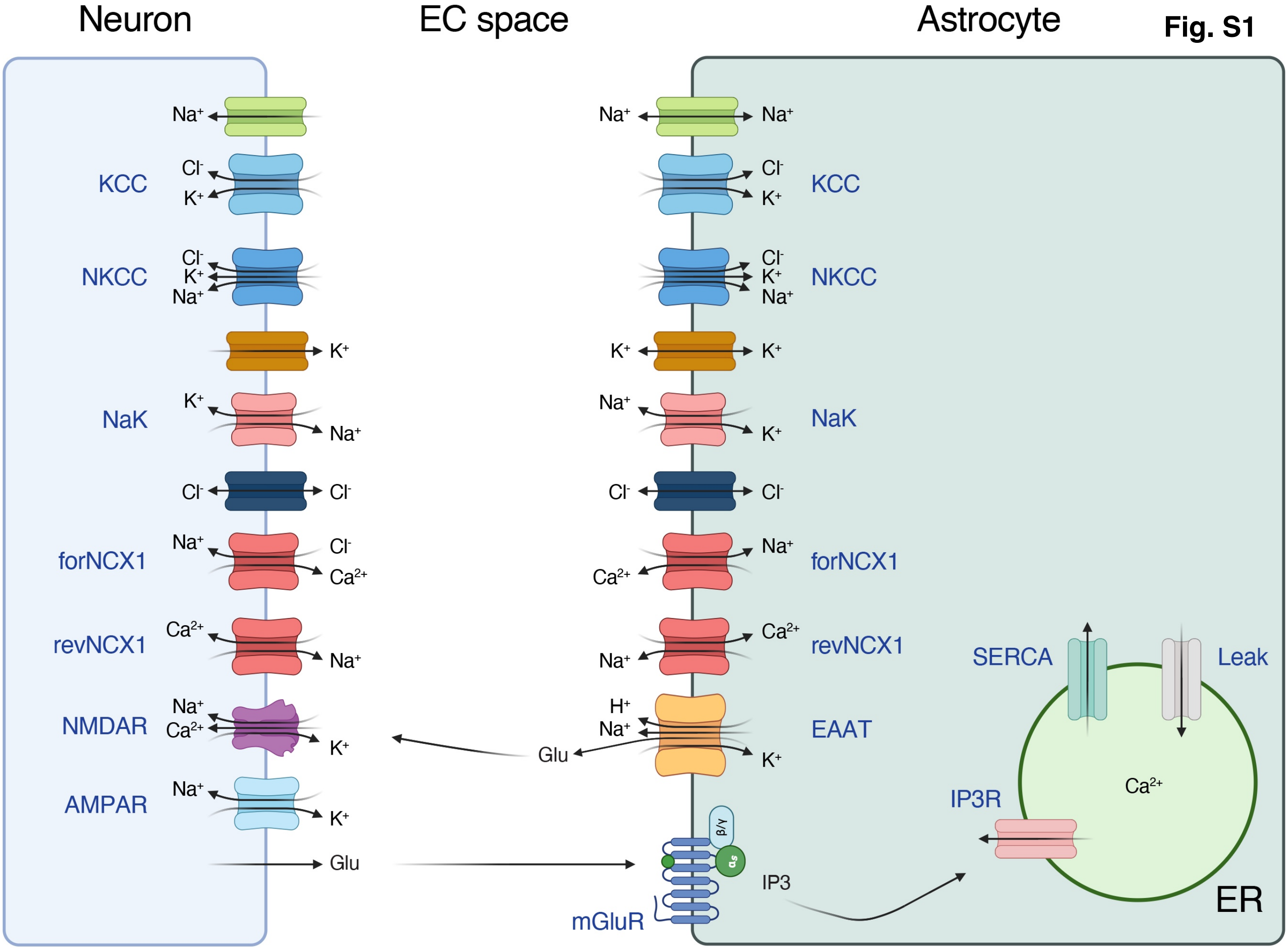
Neuron
EC space
Astrocyte
Fig. S1
Na+
Cl-
K+
KCC
NKCC
Cl-
K+
Na+
K+
NaK
K+
Na+
Cl-
Cl-
forNCX1
Na+
Cl-
Ca2+
revNCX1
Ca2+
Na+
NMDAR
Na+
Ca2+
K+
AMPAR
Na+
K+
Glu
Glu
Na+
Na+
Cl-
K+
KCC
Cl-
K+
Na+
NKCC
K+
K+
Na+
K+
NaK
Cl-
Cl-
Ca2+
Na+
forNCX1
Na+
Ca2+
revNCX1
H+
Na+
K+
EAAT
β/γ
αs
IP3
mGluR
SERCA
Leak
Ca2+
IP3R
ER
